# Burden of cancer in Africa, 1990–2023: a systematic analysis from the Global Burden of Disease Study 2023

**DOI:** 10.1016/S1470-2045(26)00291-3

**Published:** 2026-10

**Authors:** GBD 2023 Africa Cancer Risk Factors Collaborators, Atalel Fentahun Awedew, Sherief Abd-Elsalam, Auwal Abdullahi, Mesfin Abebe, Kedir Hussein Abegaz, Alemwork Abie, Richard Gyan Aboagye, Bilyaminu Abubakar, Rufus Adesoji Adedoyin, Ayman Ahmed, Timothy Olukunle Aladelusi, Mohammed Usman Ali, Hubert Amu, Ganiyu Adeniyi Amusa, Haftu Asmerom, Amlaku Mulat Aweke, Ahmed Y Azzam, Wondu Feyisa Feyisa Balcha, Afisu Basiru, Nebiyou Simegnew Bayleyegn, Bekalu Mekonen Belay, Melesse Belayneh, Alemshet Yirga Berhie, Eyob Ketema Bogale, Souad Bouaoud, Isaac Sunday Chukwu, Bashir Dabo, Robert Kokou Dowou, Temitope Cyrus Ekundayo, Iman El Sayed, Muhammed Elhadi, Mohammed Elshaer, Ibrahim Elsohaby, Destaw Endeshaw, Omotayo Francis Fagbule, Ginenus Fekadu, Bikila Regassa Feyisa, Muktar A Gadanya, Mesfin Gebrehiwot, Genanew K Getahun, Simegnew Asmer Getie, Alem Abera Girmay, Mesay Dechasa Gudeta, Tesfahun Simon Simon Hadaro, Nadia M Hamdy, Ahmed I Hasaballah, Ikrama Hassan, Mohamed Hsairi, Segun Emmanuel Ibitoye, Paul Chukwudi Ikwegbue, Mustapha Immurana, Faisal Ismail, Chidozie Declan Iwu, Alelign Tasew Jema, Kehinde Kazeem Kanmodi, Basira Kankia Lawal, Tesfahun Mekene Meto, Hadush Negash Meles, Endalkachew Worku Mengesha, Ahmed Ismail Mohamed, Jama Mohamed, Nouh Saad Mohamed, Shafiu Mohammed, Rawlance Ndejjo, Amanuel Tebabal Nega, Chakib Nejjari, Chukwudi A Nnaji, Chisom Adaobi Nri-Ezedi, Ismail A Odetokun, Akinyemi O D Ofakunrin, Ropo Ebenezer Ogunsakin, Akinkunmi Paul Okekunle, Hany A Omar, Abidemi E Omonisi, Maureene Auma Ondayo, Obinna E Onwujekwe, Mayowa O Owolabi, Oyetunde T Oyeyemi, Nasiru Raheem, Elrashdy M. Redwan, Mohamed A Saleh, Aminu Shittu, Emmanuel Edwar Siddig, Iman M Talaat, Jacques Lukenze Tamuzi, Birhan Tsegaw Taye, Dufera Rikitu Terefa, Wegen Beyene Tesfamariam, Musliu Adetola Tolani, Aniefiok John Udoakang, Shehu Salihu Umar, Gizachew Tadesse Wassie, Zwanden Sule Yahaya, Subah Abderehim Yesuf, Mohammed G M Zeariya, Abdulaziz Mohammed, Ali H Mokdad, Awoke T Misganaw

**Affiliations:** A Department of Surgery, Debre Tabor University, Debre Tabor, Ethiopia; B Department of Tropical Medicine and Infectious Diseases, Tanta University, Egypt, Tanta, Egypt; C Department of Physiotherapy, Bayero University Kano, Kano, Nigeria; D Department of Physiotherapy, Federal University Wukari, Wukari, Nigeria; E Department of Midwifery, Dilla University, Dilla, Ethiopia; F Department of Biostatistics, Madda Walabu University, Bale Robe, Ethiopia; G Department of Midwifery, Bahir Dar University, Bahir Dar, Ethiopia; H Department of Family and Community Health, University of Health and Allied Sciences, Ho, Ghana; I School of Population Health, University of New South Wales, Sydney, NSW, Australia; J Department of Pharmacology and Toxicology, Usmanu Danfodiyo University, Sokoto, Sokoto, Nigeria; K Nigerian Institute of Medical Research, Lagos, Nigeria; L Department of Medical Rehabilitation, Obafemi Awolowo University, Ile-Ife, Nigeria; M Institute of Endemic Diseases, University of Khartoum, Khartoum, Sudan; N Pan-African One Health Institute (PAOHI), Kigali, Rwanda; O Department of Oral and Maxillofacial Surgery, University of Ibadan, Ibadan, Nigeria; P Department of Oral and Maxillofacial Surgery, University College Hospital, Ibadan, Ibadan, Nigeria; Q Department of Medical Rehabilitation (Physiotherapy), University of Maiduguri, Maiduguri, Nigeria; R Nethersole School of Nursing, The Chinese University of Hong Kong, Hong Kong, China; S Department of Population and Behavioural Sciences, University of Health and Allied Sciences, Ho, Ghana; T Department of Medicine, University of Jos, Jos, Nigeria; U Department of Internal Medicine, Jos University Teaching Hospital, Jos, Nigeria; V School of Medical Laboratory Sciences, Haramaya University, Harar, Ethiopia; W ASIDE Healthcare, Lewes, DE, USA; X Faculty of Medicine, October 6 University, 6th of October City, Egypt; Y Department of Veterinary Physiology and Biochemistry, University of Ilorin, Ilorin, Nigeria; Z Department of Surgery, Jimma University, Jimma, Ethiopia; AA Department of Nursing, Debre Tabor University, Debre Tabor, Ethiopia; AB Department of Public Health, Bahir Dar University, Bahir Dar, Ethiopia; AC Department of Public Health, University of South Africa, Pretoria, South Africa; AD Department of Nursing, Bahir Dar University, Bahir Dar, Ethiopia; AE Department of Health Promotion and Behavioural Science, Bahir Dar University, Bahir Dar, Ethiopia; AF Department of Medicine, University Ferhat Abbas of Setif, Setif, Algeria; AG Department of Epidemiology and Preventive Medicine, University Hospital Saadna Abdenour, Setif, Algeria; AH Department of Paediatric Surgery, Federal Medical Centre, Umuahia, Nigeria; AI Department of Epidemiology and Biostatistics, University of South Florida, Tampa, FL, USA; AJ Department of Medical Microbiology and Parasitology, Bayero University, Kano, Nigeria; AK Department of Epidemiology and Biostatistics, University of Health and Allied Sciences, Ho, Ghana; AL Department of Microbiology, University of Medical Sciences, Ondo, Ondo, Nigeria; AM Biomedical Informatics and Medical Statistics Department, Alexandria University, Alexandria, Egypt; AN College of Medicine, Korea University, Seoul, South Korea; AO Houston Methodist Hospital, Houston, TX, USA; AP Department of Clinical Pathology, Mansoura University, Mansoura, Egypt; AQ Department of Infectious Diseases and Public Health, City University of Hong Kong, Hong Kong, China; AR Department of Animal Medicine, Zagazig University, Zagazig, Egypt; AS Department of Adult Health Nursing, Bahir Dar University, Bahir Dar, Ethiopia; AT Department of Periodontology and Community Dentistry, University of Ibadan, Ibadan, Nigeria; AU School of Public Health, University of Nevada Reno, Reno, NV, USA; AV Department of Pharmacy, Wollega University, Nekemte, Ethiopia; AW Institute of Health Sciences, Wollega University, Nekemte, Ethiopia; AX Jimma University, Jimma, Ethiopia; AY Department of Community Medicine, Bayero University Kano, Kano, Nigeria; AZ Department of Community Medicine, Aminu Kano Teaching Hospital, Kano, Nigeria; BA Environmental Pollution Monitoring and Study Desk, Ethiopian Environmental Protection Authority, Addis Ababa, Ethiopia; BB Department of Public Health, Menelik II Medical and Health Science College, Addis Ababa, Ethiopia; BC Department of Nursing, Aksum University, Aksum, Ethiopia; BD Department of Clinical Pharmacy, Haramaya University, Harar, Ethiopia; BE Department of Midwifery, Arba Minch University, Arba Minch, Ethiopia; BF Biochemistry and Molecular Biology Department, Ain Shams University, Cairo, Egypt; BG Department of Zoology and Entomology, Al-Azhar University, Cairo, Egypt; BH Department of Community Medicine, Federal University Teaching Hospital, Lafia, Nigeria; BI Department of Epidemiology and Community Medicine, Federal University of Lafia, Lafia, Nigeria; BJ Faculty of Medicine of Tunis, University Tunis El Manar, Tunis, Tunisia; BK Department of Health Promotion and Education, University of Ibadan, Ibadan, Nigeria; BL Department of Pathology, University of Cape Town, Cape Town, South Africa; BM Institute of Health Research, University of Health and Allied Sciences, Ho, Ghana; BN Clinical Laboratory Department, Tobruk University, Tobruk, Libya; BO Department of Blood Transmitted Diseases, National Centre for Disease Control (NCDC), Tobruk, Libya; BP School of Health Systems and Public Health, University of Washington, Seattle, WA, USA; BQ Department of Public Health, Madda Walabu University, Goba, Ethiopia; BR Office of the Executive Director, Cephas Health Research Initiative Inc., Ibadan, Nigeria; BS Department of Public Health, Thomas Adewumi University, Oko, Nigeria; BT Department of Clinical Pharmacy and Pharmacy Management, Kaduna State University, Kaduna, Nigeria; BU Department of Public Health, Arba Minch University, Arba Minch, Ethiopia; BV Department of Medical Laboratory Sciences, Adigrat University, Adigrat, Ethiopia; BW Department of Reproductive Health and Population Studies, Bahir Dar University, Bahir Dar, Ethiopia; BX College of Health Science, University of Hargeisa, Hargeisa, Somalia; BY Institute of Health Science, Jimma University, Jimma, Ethiopia; BZ College of Applied and Natural Science, University of Hargeisa, Hargeisa, Somalia; CA Molecular Biology Unit, Sirius Training and Research Centre, Khartoum, Sudan; CB Bio-Statistical and Molecular Biology Department, Sirius Training and Research Centre, Khartoum, Sudan; CC Health Systems and Policy Research Unit, Ahmadu Bello University, Zaria, Nigeria; CD Heidelberg Institute of Global Health (HIGH), Heidelberg University, Heidelberg, Germany; CE Department of Disease Control and Environmental Health, Makerere University, Kampala, Uganda; CF Euromed Research Center, Euromed University of Fes, Fez, Morocco; CG Faculty of Medicine, Pharmacy, and Dentistry, University Sidi Mohammed Ben Abdellah, Fez, Morocco; CH Technical Department, University of Cape Town, Cape Town, South Africa; CI School of Public Health and Family Medicine, University of Cape Town, Cape Town, South Africa; CJ Department of Paediatrics, Nnamdi Azikiwe University, Awka, Nigeria; CK Department of Veterinary Public Health and Preventive Medicine, University of Ilorin, Ilorin, Nigeria; CL Department of Pediatrics, University of Jos, Jos, Nigeria; CM Department of Pediatrics, Jos University Teaching Hospital, Jos, Nigeria; CN School of Health Systems & Public Health, University of Pretoria, Pretoria, South Africa; CO Department of Food and Nutrition, Seoul National University, Seoul, South Korea; CP College of Medicine, University of Ibadan, Ibadan, Nigeria; CQ Department of Pharmacy Practice and Pharmacotherapeutics, University of Sharjah, Sharjah, United Arab Emirates; CR Department of Pharmacology and Toxicology, Beni-Suef University, Beni-Suef, Egypt; CS Department of Anatomic Pathology, Ekiti State University, Ado- Ekiti, Nigeria; CT Department of Anatomic Pathology, Ekiti State University Teaching Hospital, Ado Ekiti, Nigeria; CU Department of Environmental Biology and Health, University of Eldoret, Eldoret, Kenya; CV Department of Environmental Health and Biology, University of Eldoret, Eldoret, Kenya; CW Department of Pharmacology and Therapeutics, University of Nigeria Nsukka, Enugu, Nigeria; CX Department of Medicine, University of Ibadan, Ibadan, Nigeria; CY Department of Medicine, University College Hospital, Ibadan, Ibadan, Nigeria; CZ Department of Biosciences and Biotechnology, University of Medical Sciences, Ondo, Ondo, Nigeria; DA Department of Anatomic Pathology and Forensic Medicine, Modibbo Adama University Teaching Hospital, Yola, Nigeria; DB Department of Biological Sciences, King Abdulaziz University, Jeddah, Egypt; DC Department of Protein Research, Research and Academic Institution, Alexandria, Egypt; DD College of Medicine, University of Sharjah, Sharjah, United Arab Emirates; DE Faculty of Pharmacy, Mansoura University, Mansoura, Egypt; DF Department of Veterinary Public Health and Preventive Medicine, Usmanu Danfodiyo University, Sokoto, Sokoto, Nigeria; DG Unit of Basic Medical Sciences, University of Khartoum, Khartoum, Sudan; DH Department of Medical Microbiology and Infectious Diseases, Erasmus University, Rotterdam, Netherlands; DI Clinical Sciences Department, University of Sharjah, Sharjah, United Arab Emirates; DJ Department of Pathology, Alexandria University, Alexandria, Egypt; DK Department of Epidemiology, Stellenbosch University, Cape Town, South Africa; DL Department of Medicine, Northlands Medical Group, Omuthiya, Namibia; DM School of Nursing and Midwifery, Debre Berhan University, Debre Berhan, Ethiopia; DN Outpatient Department, Wollega University, Bedele Town, Ethiopia; DO Department of Public Health, Wollega University, Nekemte, Ethiopia; DP College of Health Sciences, Mekelle University, Mekelle, Ethiopia; DQ Department of Surgery, Ahmadu Bello University, Zaria, Nigeria; DR Department of Oncology, Federal Medical Centre, Gusau, Nigeria; DS Department of Community Medicine, Ahmadu Bello University, Zaria, Nigeria; DT Department of Epidemiology and Biostatistics, Bahir Dar University, Bahir Dar, Ethiopia; DU Department of Pharmaceutics and Industrial Pharmacy, Kaduna State University, Kaduna, Nigeria; DV Department of Family Medicine, St. Paul's Hospital Millennium Medical College, Addis Ababa, Ethiopia; DW Family Medicine Department, St. Peter's Specialized Hospital, Addis Ababa, Ethiopia; DX Department of Public Health, University of Hail, Hail, Saudi Arabia; DY Department of Disease Control and Prevention, Africa Centre for Disease Control and Prevention, Addis Ababa, Ethiopia; DZ Institute for Health Metrics and Evaluation, University of Washington, Seattle, WA, USA; EA Department of Health Metrics Sciences, School of Medicine, University of Washington, Seattle, WA, USA; EB National Data Management Center for Health (NDMC), Ethiopian Public Health Institute, Addis Ababa, Ethiopia

## Abstract

**Background:**

Cancer remains a leading cause of death and disability worldwide, with a disproportionately rising burden across Africa. Effective policy responses have been consistently hampered by fragmented and incomplete cancer surveillance data. Using the Global Burden of Diseases, Injuries, and Risk Factors Study (GBD) 2023, we estimated the burden of 48 cancer types across all 54 African countries over the period 1990 to 2023. These age-disaggregated and sex-disaggregated findings provide a crucial empirical foundation to inform regional control strategies and monitor progress towards global health targets.

**Methods:**

We applied GBD 2023 methodology to estimate fatal and non-fatal outcomes of cancer alongside risk-attributable burden from 88 modifiable risk factors, across all 54 African countries. The estimation of cancer burden attributable to risk factors followed the comparative risk assessment framework. Estimates of incidence, mortality, years lived with disability (YLDs), years of life lost (YLLs), and disability-adjusted life-years (DALYs) were stratified by age, sex, location, and year (1990–2023) and reported as counts and age-standardised rates per 100 000 population. Mean estimates represent the mean across 250 draws, with 95% uncertainty intervals calculated from the 2·5th and 97·5th percentiles.

**Findings:**

In 2023, cancer, including non-melanoma skin cancer, accounted for an estimated 1·54 million (95% uncertainty interval 1·37–1·75) incident cases, 923 000 (824 000–1 020 000) deaths, and 33·9 million (30·6–37·1) DALYs across all ages and both sexes in Africa, of which 98·2% (97·9–98·3) were attributable to YLLs. In Africa, 10·7 million (8·9–12·9) DALYs and 306 000 (254 000–363 000) cancer deaths in 2023 were attributable to the 88 risk factors assessed in GBD 2023, equivalent to 31·6% (26·8–35·9) of total DALYs and 33·1% (28·7–37·2) of total cancer deaths across all ages and both sexes. Between 1990 and 2023, deaths attributable to risk factors increased by 216·0% (159·3–284·5) for both sexes combined. In 2023, incident cancer cases in females numbered 960 000 (821 000–1 130 000), with breast and cervical cancers together accounting for 53·5% (44·7–62·2) of all female incident cases. In males, prostate, liver, tracheal, bronchus and lung, and colorectal cancers were the leading types, together accounting for 44·8% (36·2–54·3) of all male cancers in Africa in 2023. Childhood cancer (ages 0–19 years) in Africa accounted for 104 000 (84 600–130 000) incident cases and 54 700 (47 500–64 500) deaths, representing 27·7% (26·6–29·4) of global incidence and 38·0% (36·3–40·0) of global deaths in 2023. Cancers in adolescents and young adults (ages 15–39 years) contributed 26·6% (26·4–27·5) of total cancer DALYs in Africa, those in older adults aged 40–64 years contributed 44·8% (43·8–45·6), and those in adults aged 65 years and older contributed 18·3% (17·4–19·1).

**Interpretation:**

Cancer is a growing contributor to the disease burden in Africa, with rising cases, deaths, and DALYs disproportionately affecting children, adolescents, and young adults. The increase in age-standardised mortality and DALY rates stands in sharp contrast to the ambitions of the Sustainable Development Goals for 2030 and the African Union Agenda 2063. Addressing this burden will require coordinated national and international action, with health-system strengthening and context-specific cancer-control strategies spanning prevention, early diagnosis, and treatment.

**Funding:**

Gates Foundation.

## Introduction

Cancer is one of the top global public health issues, accounting for a sizable portion of mortality, morbidity, disability, and economic costs worldwide.^1^, ^2^ Cancer is a substantial public health concern in Africa, ranking as one of the top three causes of premature death among individuals aged 30–69 years in nearly all countries on the continent.^3^, ^4^ It accounts for one in seven premature deaths overall and one in four deaths from non-communicable diseases (NCDs).^4^ The burden of cancer in Africa has been increasing in the past three decades as a result of increasing population size, ageing, substantially decreased mortality from other causes, and epidemiological and demographic transitions worldwide.^2^, ^4^, ^5^, ^6^, ^7^ Despite the rising global burden of cancer, the effects of cancer are inequitably distributed; the worst effects are in low-income countries in Africa.^1^ According to WHO forecasts, low-income and middle-income countries (LMICs) will have more than 67% of the global burden of cancer in 2040.^1^, ^8^ These discrepancies are due to inequities in the distribution of cancer risk factors, accessibility of cancer prevention measures, early detection, treatment, care, rehabilitation, and quality of life after diagnosis of cancer.^9^ Global evidence on cancer indicates that the annual burden in Africa will nearly double by 2045, reaching an estimated 1·4 million new cases and 1 million deaths.^4^, ^10^

**Research in context**

**Evidence before this study**

We searched PubMed for articles published between Jan 1, 1990, and Dec 31, 2025, using the terms “Africa” AND (“DALYs”, “incidence”, “death” OR “burden of cancer”). Our search identified Global Burden of Diseases, Injuries, and Risk Factors Study (GBD) and GLOBOCAN analyses that primarily focused on sub-Saharan Africa. No study to date has synthesised GBD data across all 54 African countries, covering all cancer types and stratified by demographic and temporal dimensions. This gap highlights the need for a comprehensive analysis to inform regional and national cancer control strategies.

**Added value of this study**

This study provides a comprehensive, continent-wide assessment of cancer-related health loss in Africa, using the GBD 2023 study to quantify the impact of 88 risk factors across all 54 countries. By characterising geographical and temporal variations, our findings reveal substantial spatiotemporal heterogeneity and epidemiological patterns that diverge from global trends—most notably the disproportionate burden among children, adolescents, and young adults. We estimate that about a third of cancer-related mortality and DALYs in the region are attributable to modifiable risk factors. Furthermore, Africa accounts for nearly 38% of global childhood cancer cases, positioning the continent at the epicenter of global childhood oncology. These results establish a robust, unified evidence base for understanding the scale and determinants of cancer in Africa, advancing beyond fragmented national reporting to enable high-resolution, comparative insights across populations and regions.

**Implications of all the available evidence**

Cancer is a major contributor to the disease burden in Africa, characterised by escalating age-standardised mortality and DALY rates that contrast sharply with declining trends in global and high-income settings. A substantial proportion of this burden is attributable to modifiable risk factors, presenting significant opportunities for mitigation through targeted prevention efforts. Crucially, the cancer burden in Africa disproportionately affects children, adolescents, and young adults, a finding that diverges from global epidemiological patterns. These results are essential for evaluating progress towards SDG 3.4, the African Union Agenda 2063, and other international health targets. As cancer burden is projected to rise in the coming decades, addressing this inequitable growth will require comprehensive and sustainable national and international efforts that integrate context-specific health system considerations into the development and implementation of cancer-control strategies.

Global initiatives, including WHO programmes for cervical,^11^ breast,^12^ and childhood^13^ cancers, alongside *Lancet Oncology* Commissions,^7^, ^14^ have delineated clear strategies to mitigate the burgeoning cancer burden in LMICs. These objectives are congruent with Sustainable Development Goal (SDG) 3.4^15^ and the African Union's Agenda 2063,^16^ both of which prioritise the reduction of premature mortality from cancer. Regional efforts are further bolstered by the Africa Centres for Disease Control and Prevention (CDC) Strategic Plan 2023–27, which emphasises integrated surveillance and health system resilience.^17^

Accurate data on cancer burden are essential for resource allocation and to monitor progress towards global and regional targets. Yet, surveillance systems remain scarce or absent in many African countries. To address these gaps, two major initiatives routinely generate estimates: the Global Burden of Diseases, Injuries, and Risk Factors Study (GBD) and GLOBOCAN from the International Agency for Research on Cancer. Both rely on existing data sources to model cancer burden where direct data are limited and fragmented, but differ in methodology and scope. GBD provides expanded measures—including incidence, prevalence, mortality, years of life lost (YLLs), years lived with disability (YLDs), and disability-adjusted life-years (DALYs)—and includes cancers in children, adolescent, and young adult populations, whereas GLOBOCAN focuses primarily on incidence and mortality.

Despite these efforts, the paucity of granular, continent-wide data remains a major barrier to effective policy. This study quantifies incidence, mortality, and DALYs across 54 African nations, disaggregated by age, sex, location, and year, to inform targeted interventions and regional cancer control strategies. This manuscript was produced as part of the GBD Collaborator Network and in accordance with the GBD Protocol.

## Methods

### Study overview

GBD is an evolving research effort that quantifies the state of global health. For each new GBD iteration, recently available data and improved methods are used to update the full time series of estimates from 1990 to the latest year of analysis. GBD 2023 estimates therefore supersede all previous estimates. GBD 2023 produced estimates for each epidemiological quantity of interest for 376 diseases and injuries—including 294 causes of death—and 88 risk factors by age, sex, location, and year for 25 age groups from birth to age 95 years and older; for males, females, and all sexes combined; in 204 countries and territories grouped into 21 regions and seven super-regions, as well as subnational locations in 20 countries and territories.^18^, ^19^ The present analysis focuses on 54 African countries: six from the GBD super-region north Africa and the Middle East, two from southeast Asia, east Asia, and Oceania, and 46 from sub-Saharan Africa. We report incidence, deaths, DALYs and YLLs, and risk-attributable burden stratified by age, sex, location, and year.^18^, ^19^ Headline totals are reported for the GBD total cancers aggregate, which includes non-melanoma skin cancer; cause-specific estimates are presented for 47 GBD cancer groups plus non-melanoma skin cancer separately; other neoplasms is excluded throughout. The study adheres to the Guidelines for Accurate and Transparent Health Estimates Reporting (GATHER), ensuring transparency and methodological rigour.^20^

### Data sources

The GBD 2023 estimates for fatal and non-fatal cancer burden were derived from 4499 population-based registries.^18^, ^19^, ^21^ Incidence and mortality data were sourced from major international programmes, including the Surveillance, Epidemiology, and End Results (SEER) programme, Cancer Incidence in Five Continents (CI5), EUREG, and NORDCAN, and 70 population-based registries in Africa.^21^ The analysis further used 19 485 site-years of vital registration data and 421 site-years of data from verbal autopsy sources.^21^ Comprehensive source details are provided in the appendix (pp 24–52).

### Mortality estimation

The GBD study estimates cancer mortality using a comprehensive data processing workflow that ensures accuracy and consistency.^18^, ^19^, ^21^ The process begins with data collection from various sources, including cancer registries, vital registration systems, verbal autopsies, and census or survey data.^18^, ^19^, ^22^ This initial step involves standardising the collected files to ensure uniformity across formats, categorisation, and naming conventions. Next, cancer registry data are mapped to GBD causes, standardised to GBD age groups, and redistributed to specific GBD causes of death. The processed data are then matched to generate mortality-to-incidence ratios (MIRs). These ratios are incorporated into a three-step modelling approach, using spatiotemporal Gaussian process regression (ST-GPR) and incorporating the Healthcare Access and Quality (HAQ) Index as a covariate. The final MIRs are used to generate mortality estimates, which are subsequently smoothed using a Bayesian noise-reduction algorithm to address zero count issues. To account for the complex interplay of factors influencing causes of death, the GBD uses a cause of death ensemble model (CODEm). CODEm uses various statistical models and selects the most robust combination based on out-of-sample predictive validity. The resulting mortality estimates are further refined using CoDCorrect, an algorithm that ensures consistency between individual cause-level mortality and overall mortality.^18^, ^19^, ^22^ Finally, YLLs are calculated by multiplying final death estimates by the standard GBD 2023 life expectancy given the age at death, sex, and location.^21^ Detailed descriptions of data sources, processing methods, and modelling approaches are available in GBD 2023 and previous GBD publications^18^, ^19^, ^21^, ^23^ and the appendix (pp 1–102).

### Non-fatal outcome estimation

The GBD 2023 study used a sophisticated modelling approach to estimate cancer prevalence and incidence across the African continent.^21^ DisMod-MR 2.1, a Bayesian disease modelling meta-regression tool, was used to generate internally consistent estimates of prevalence, incidence, remission, and mortality for each sex, location, year, and age group. This powerful tool fills data gaps by estimating prevalence across a hierarchical structure, leveraging data from locations higher in the hierarchy to inform estimates for locations with limited data. For certain causes, the study used ST-GPR models, a set of regression methods designed to analyse heterogeneous and incomplete data, smoothing estimates across time, age, and location. The study begins by estimating cancer incidence using a two-step approach. First, adjusted GBD cancer mortality estimates are transformed into incidence estimates using cancer-specific mortality-to-incidence ratios. This process generates 1000 incidence estimates with 95% uncertainty intervals (UIs), assuming independence between MIRs and mortality uncertainties. Next, the study estimates cancer prevalence by combining incidence estimates with annual relative survival estimates (1–10 years after diagnosis). Relative survival is estimated using cancer-specific models trained on data from the SEER programme. These models predict 5-year survival from MIRs, and then generate yearly survival estimates up to 10 years using SEER data and proportional scaling based on GBD predictions. Finally, relative survival is converted to absolute survival using GBD 2021 life tables and an exponential survival function, allowing for the calculation of prevalence at each year after diagnosis. To understand the disease progression, the study divides the prevalence into four distinct stages or sequelae: diagnosis and primary therapy phase; controlled phase (remission); disseminated or metastatic phase; and terminal phase. To estimate the burden of cancer procedures, hospital data were used to quantify the number of patients undergoing procedures and assess procedure-related disability. Last, the procedure sequelae prevalence and general sequelae prevalence were multiplied with their respective disability weights to obtain the number of YLDs for each sequela. Detailed descriptions of data sources, processing methods, and modelling approaches are available in GBD 2023^18^, ^19^, ^21^ and previous GBD publications^22^, ^24^, ^25^, ^26^ and the appendix (pp 1–102).

### Risk factor estimation

The estimation of cancer burden attributable to risk factors followed the comparative risk assessment framework. In total, 88 risk factors were assessed using four key inputs: exposure, relative risk, the theoretical minimum risk exposure level, and the burden of deaths and disability linked to each outcome. Exposure estimation drew on more than 55 000 distinct data sources, including approximately 16 000 newly incorporated for GBD 2023. Risk factor analysis was stratified by age, sex, location, and year. Population attributable fractions were computed for each risk–outcome pair, with mediation adjustments applied where risks act through intermediate pathways. In GBD 2023, 159 health outcomes were linked to risk factors through 676 risk–outcome pairs, including 50 newly added pairs. Detailed methods are provided in GBD 2023.^18^, ^21^, ^24^

### Uncertainty estimation

Mean estimates for all other metrics reported represent the mean value across 250 draws from the estimate's distribution, with 95% UIs calculated as the 2·5th and 97·5th percentile values across the draws.^18^, ^19^, ^21^

### Role of the funding source

The funder of the study had no role in study design, data collection, data analysis, data interpretation, or the writing of the report.

## Results

In Africa, incident cancer cases rose from 467 000 (95% UI 420 000–528 000) in 1990 to 808 000 (726 000–917 000) in 2010 and 1·54 million (1·37–1·75) in 2023 (1·46 million [1·30–1·67] when excluding non-melanoma skin cancer; GBD results tool), corresponding to relative increases of 90·8% (64·7–116·1) between 2010 and 2023 and 229·0% (183·5–286·2) between 1990 and 2023 across all ages and both sexes. Over the same period, the age-standardised incidence rate rose from 131·2 (118·0–147·3) per 100 000 in 1990 to 137·4 (123·8–154·2) per 100 000 in 2010 and 172·1 (154·1–193·4) per 100 000 in 2023, corresponding to relative increases of 25·2% (9·2–40·6) between 2010 and 2023 and 31·1% (13·6–51·3) between 1990 and 2023. In 2023, incident cancer cases were higher in females (960 000 [821 000–1 130 000]) than in males (580 000 [491 000–667 000]; table 1; appendix pp 104–115).

**Table 1:** Cancer incidence, death, and DALY counts and age-standardised rates in 2023 and percentage change in counts and age-standardised rates between 1990 and 2023 in Africa for all ages and sexes combined by country

			**Incidence**				**Deaths**				**DALYs**			
			Cases, thousands, 2023	Age-standardised rate, 2023	Percentage change in cases, 1990–2023	Percentage change in age-standardised rate, 1990–2023	Count, thousands, 2023	Age-standardised rate, 2023	Percentage change in count, 1990–2023	Percentage change in age-standardised rate, 1990–2023	Count, ten thousands, 2023	Age-standardised rate, 2023	Percentage change in count, 1990–2023	Percentage change in age-standardised rate, 1990–2023
Africa			1540 (1370 to 1750)	172·1 (154·1 to 193·4)	229·0% (183·5 to 286·2)	31·1% (13·6 to 51·3)	923 (824 to 1020)	114·2 (100·6 to 125·8)	185·0% (151·9 to 219·4)	15·5% (2·3 to 31·9)	3390 (3060 to 3710)	3443·1 (3092·9 to 3781·0)	162·5% (128·5 to 194·9)	11·5% (−1·6 to 24·6)
North Africa*			302 (250 to 365)	151·4 (128·7 to 176·1)	275·2% (212·7 to 353·1)	47·0% (23·0 to 77·4)	179 (149 to 213)	96·2 (83·5 to 109·6)	208·2% (156·4 to 272·5)	18·7% (−2·9 to 44·3)	548 (461 to 648)	2588·9 (2241·9 to 2931·2)	165·8% (123·7 to 212·7)	13·9% (−4·3 to 35·9)
	Algeria		51·7 (41·1 to 65·5)	122·0 (97·0 to 154·2)	268·4% (192·1 to 362·9)	35·7% (10·5 to 69·3)	24·5 (19·1 to 30·3)	62·6 (47·9 to 77·6)	189·3% (135·9 to 240·1)	2·1% (−16·7 to 20·8)	78·7 (63·3 to 95·0)	1829·2 (1466·7 to 2220·7)	126·3% (86·1 to 168·5)	−5·1% (−21·8 to 10·6)
	Egypt		121 (103 to 141)	164·6 (141·0 to 191·6)	266·6% (196·0 to 362·3)	53·1% (21·4 to 94·2)	78·3 (66·9 to 91·1)	119·1 (101·0 to 138·9)	216·2% (153·3 to 302·7)	33·1% (5·3 to 73·1)	239 (206 to 276)	3004·2 (2562·7 to 3499·6)	173·2% (123·8 to 240·9)	22·2% (−1·7 to 54·7)
	Libya		11·0 (9·20 to 13·2)	166·2 (137·3 to 198·9)	246·4% (171·7 to 340·4)	20·1% (−3·7 to 50·4)	5·90 (4·90 to 7·10)	99·5 (82·2 to 119·5)	185·6% (133·4 to 241·1)	0·3% (−17·3 to 20·9)	19·8 (16·5 to 23·4)	2860·0 (2382·0 to 3398·3)	157·6% (111·3 to 204·6)	−3·4% (−20·2 to 15·4)
	Morocco		50·4 (40·6 to 63·3)	131·5 (106·4 to 164·4)	328·5% (250·7 to 424·8)	73·4% (42·0 to 114·8)	29·6 (24·6 to 36·6)	81·6 (67·8 to 101·4)	240·7% (174·5 to 314·5)	34·0% (7·9 to 62·4)	89·1 (73·8 to 110)	2290·6 (1905·4 to 2852·0)	211·7% (151·5 to 279·9)	38·2% (10·9 to 67·0)
	Sudan		34·8 (28·4 to 43·2)	136·7 (111·8 to 169·0)	187·3% (136·1 to 253·2)	20·8% (−1·0 to 46·6)	23·0 (18·0 to 29·1)	99·9 (77·3 to 126·5)	144·4% (98·8 to 196·7)	2·4% (−17·4 to 26·1)	74·1 (58·8 to 92·0)	2666·9 (2108·9 to 3319·7)	112·5% (76·9 to 161·1)	0·3% (−16·4 to 21·4)
	Tunisia		33·1 (28·0 to 38·6)	238·5 (199·7 to 279·2)	258·3% (194·2 to 344·7)	33·2% (10·2 to 66·5)	17·4 (15·6 to 19·2)	129·8 (115·5 to 143·6)	166·1% (127·1 to 215·0)	−6·7% (−20·6 to 11·2)	47·0 (42·8 to 51·4)	3356·9 (3052·7 to 3673·0)	134·3% (101·8 to 174·1)	−4·7% (−18·5 to 11·7)
Mauritius†			2·90 (2·60 to 3·20)	158·9 (142·7 to 176·3)	185·8% (148·8 to 228·9)	23·2% (8·2 to 40·8)	1·70 (1·60 to 1·80)	92·9 (87·8 to 97·6)	143·9% (129·2 to 159·5)	−2·7% (−7·8 to 3·4)	4·40 (4·20 to 4·70)	2469·8 (2358·7 to 2586·5)	112·8% (98·8 to 126·2)	−3·0% (−8·9 to 2·9)
Seychelles†			0·300 (0·300 to 0·300)	240·6 (211·9 to 275·9)	176·9% (131·5 to 234·9)	27·1% (6·3 to 53·6)	0·200 (0·200 to 0·200)	146·0 (133·0 to 158·6)	122·2% (93·6 to 153·9)	3·5% (−10·2 to 18·4)	0·500 (0·500 to 0·500)	3899·4 (3617·4 to 4220·1)	121·3% (95·6 to 149·2)	1·4% (−10·7 to 14·3)
Sub-Saharan Africa			1240 (1110 to 1390)	178·4 (160·4 to 199·9)	221·9% (174·9 to 277·5)	27·6% (10·3 to 47·2)	742 (664 to 811)	119·7 (105·1 to 132·0)	182·1% (145·2 to 219·2)	15·1% (1·0 to 31·4)	2840 (2590 to 3100)	3686·3 (3313·4 to 4017·8)	163·8% (127·4 to 199·3)	10·7% (−3·9 to 25·6)
	Central sub-Saharan Africa		157 (128 to 186)	198·1 (161·6 to 231·6)	311·6% (231·3 to 399·7)	46·3% (20·1 to 76·9)	98·7 (80·1 to 114)	139·0 (111·3 to 159·9)	263·4% (199·6 to 340·5)	30·8% (7·6 to 57·8)	375 (307 to 433)	4279·2 (3468·2 to 4936·6)	249·8% (185·1 to 315·5)	32·2% (8·8 to 59·4)
		Angola	30·1 (25·0 to 34·7)	165·7 (140·3 to 189·1)	365·3% (283·8 to 478·1)	31·5% (10·7 to 64·5)	19·1 (16·2 to 22·0)	117·8 (100·5 to 134·6)	307·1% (236·8 to 402·1)	16·9% (−2·4 to 43·3)	74·5 (61·5 to 86·9)	3582·8 (3034·7 to 4114·5)	290·9% (219·7 to 380·4)	17·2% (−3·6 to 44·3)
		Central African Republic	4·80 (3·50 to 5·90)	138·7 (103·8 to 166·4)	184·7% (116·0 to 246·4)	20·4% (−4·9 to 44·8)	3·20 (2·40 to 3·90)	101·7 (76·3 to 122·0)	167·2% (113·9 to 228·7)	13·7% (−8·8 to 40·7)	12·5 (9·40 to 15·4)	3187·9 (2388·6 to 3898·2)	169·9% (110·2 to 234·8)	16·5% (−7·7 to 42·9)
		Congo (Brazzaville)	6·20 (5·00 to 7·70)	182·3 (153·3 to 217·0)	284·3% (198·7 to 387·8)	50·1% (21·2 to 85·2)	3·9 (3·2 to 4·7)	128·2 (109·8 to 152·3)	257·5% (192·8 to 345·6)	43·5% (22·3 to 75·4)	14·0 (11·3 to 17·5)	3729·6 (3092·5 to 4508·9)	229·3% (159·5 to 316·2)	31·8% (8·3 to 63·3)
		DR Congo	111 (85·8 to 136)	213·4 (167·6 to 253·9)	305·8% (218·5 to 405·5)	51·1% (20·3 to 85·4)	69·6 (54·0 to 82·4)	149·2 (114·5 to 176·4)	257·9% (183·6 to 338·0)	34·1% (6·5 to 63·1)	264 (205 to 314)	4629·9 (3606·9 to 5516·0)	244·0% (172·0 to 315·0)	37·4% (9·6 to 67·5)
		Equatorial Guinea	2·10 (1·50 to 2·60)	275·8 (218·3 to 334·8)	602·2% (413·2 to 838·4)	112·5% (61·8 to 172·3)	1·20 (0·900 to 1·40)	178·6 (143·9 to 208·1)	441·2% (314·4 to 604·1)	74·8% (37·8 to 124·7)	4·40 (3·30 to 5·40)	5388·7 (4071·3 to 6365·1)	450·6% (304·5 to 625·6)	75·0% (34·5 to 128·7)
		Gabon	3·00 (2·50 to 3·40)	212·6 (181·4 to 246·4)	288·2% (216·1 to 383·1)	68·2% (37·9 to 108·7)	1·80 (1·60 to 2·10)	138·7 (120·4 to 159·0)	234·9% (179·4 to 314·5)	46·9% (22·4 to 81·2)	6·20 (5·30 to 7·10)	4202·7 (3633·0 to 4844·2)	227·6% (163·9 to 301·1)	46·5% (19·4 to 80·0)
	Eastern sub-Saharan Africa		528 (469 to 600)	197·1 (178·5 to 223·3)	213·4% (159·7 to 276·8)	28·5% (9·1 to 52·5)	312 (285 to 339)	131·8 (117·9 to 144·8)	176·3% (142·1 to 220·3)	17·1% (2·0 to 34·2)	1260 (1160 to 1400)	4271·2 (3917·9 to 4641·0)	146·3% (111·5 to 186·3)	8·5% (−5·8 to 25·7)
		Burundi	13·6 (11·3 to 16·4)	167·1 (140·7 to 194·6)	202·6% (128·3 to 312·5)	29·8% (1·4 to 73·6)	8·10 (6·90 to 9·60)	114·7 (97·2 to 135·5)	171·6% (120·9 to 271·9)	24·1% (0·0 to 69·8)	36·3 (30·7 to 42·3)	3886·8 (3311·8 to 4586·5)	156·5% (107·6 to 248·7)	12·7% (−9·2 to 59·1)
		Comoros	0·500 (0·400 to 0·600)	78·1 (66·8 to 88·7)	129·9% (87·4 to 182·9)	−0·4% (−18·1 to 22·7)	0·300 (0·300 to 0·400)	53·0 (45·9 to 60·2)	122·6% (85·3 to 162·6)	−6·1% (−22·4 to 11·9)	1·20 (1·00 to 1·30)	1661·0 (1451·9 to 1863·9)	83·7% (54·5 to 117·1)	−13·0% (−25·7 to 4·2)
		Djibouti	1·70 (1·40 to 2·00)	164·5 (141·3 to 192·0)	417·7% (318·7 to 537·0)	10·4% (−8·9 to 33·7)	1·00 (0·900 to 1·20)	110·3 (95·4 to 127·1)	385·7% (306·1 to 481·7)	2·7% (−14·4 to 24·6)	4·00 (3·50 to 4·70)	3606·5 (3084·2 to 4140·2)	337·4% (266·7 to 425·0)	−1·6% (−17·5 to 18·0)
		Eritrea	9·70 (8·30 to 11·6)	190·3 (163·8 to 221·2)	247·5% (185·9 to 331·0)	39·6% (15·2 to 71·7)	5·80 (5·00 to 6·50)	125·1 (109·3 to 142·4)	212·4% (163·5 to 269·9)	26·6% (8·0 to 49·3)	25·2 (22·0 to 29·2)	4546·0 (3989·4 to 5195·8)	185·6% (136·0 to 244·7)	24·7% (4·3 to 48·8)
		Ethiopia	180 (150 to 216)	258·0 (221·7 to 297·6)	289·7% (212·9 to 396·3)	73·5% (43·0 to 116·0)	104 (91·9 to 116)	169·9 (147·5 to 190·5)	225·7% (169·8 to 310·7)	53·5% (27·3 to 91·3)	432 (382 to 493)	5612·0 (4996·0 to 6267·7)	169·8% (120·1 to 239·7)	32·5% (10·2 to 65·5)
		Kenya	55·7 (47·6 to 66·0)	161·7 (141·6 to 185·5)	238·2% (161·9 to 330·8)	12·4% (−10·4 to 38·4)	33·6 (29·4 to 38·0)	108·2 (94·5 to 122·8)	206·4% (150·8 to 273·0)	2·6% (−14·8 to 24·4)	127 (111 to 147)	3451·8 (3027·2 to 3937·2)	178·7% (123·8 to 241·7)	−0·3% (−19·0 to 22·4)
		Madagascar	37·2 (32·2 to 43·5)	221·4 (191·3 to 253·8)	151·8% (106·4 to 204·8)	−1·0% (−18·1 to 17·2)	23·7 (20·5 to 26·9)	163·7 (137·5 to 187·9)	132·4% (99·2 to 174·3)	−4·4% (−16·8 to 15·1)	92·7 (82·3 to 105)	4906·6 (4255·4 to 5583·7)	117·9% (83·2 to 157·4)	−13·6% (−26·3 to 2·0)
		Malawi	29·1 (22·1 to 36·7)	233·4 (184·9 to 286·6)	187·9% (107·8 to 283·8)	36·1% (1·2 to 74·4)	16·4 (13·7 to 19·6)	148·3 (124·9 to 174·8)	151·9% (96·7 to 215·6)	23·2% (−2·1 to 50·3)	70·9 (57·0 to 86·5)	5251·0 (4354·5 to 6293·8)	128·9% (75·0 to 196·5)	17·5% (−9·7 to 46·7)
		Mozambique	16·3 (12·7 to 19·7)	95·6 (78·2 to 113·8)	60·2% (20·3 to 107·7)	−16·5% (−35·2 to 6·5)	10·7 (8·60 to 12·5)	70·3 (58·3 to 82·3)	64·9% (28·4 to 110·8)	−11·2% (−29·7 to 13·8)	41·3 (32·5 to 49·5)	2037·8 (1617·9 to 2410·2)	41·5% (3·7 to 81·6)	−26·4% (−43·2 to −6·2)
		Rwanda	20·8 (18·0 to 24·3)	224·4 (195·1 to 261·3)	140·9% (93·4 to 208·8)	17·6% (−6·4 to 46·5)	12·8 (11·0 to 14·6)	153·9 (128·3 to 177·0)	123·8% (88·1 to 174·0)	12·1% (−6·2 to 36·8)	49·0 (43·1 to 55·5)	4903·7 (4236·0 to 5526·6)	77·2% (47·6 to 121·6)	−7·8% (−22·7 to 14·4)
		Somalia	11·3 (9·20 to 14·0)	92·0 (75·3 to 110·6)	233·3% (179·1 to 314·6)	17·3% (−2·1 to 43·7)	7·50 (6·20 to 8·90)	65·7 (54·7 to 78·7)	228·3% (174·1 to 305·1)	12·6% (−7·8 to 42·5)	32·6 (26·1 to 38·7)	2217·4 (1808·6 to 2662·1)	193·6% (140·5 to 264·3)	11·7% (−6·9 to 40·9)
		South Sudan	13·7 (11·5 to 16·4)	226·9 (194·9 to 263·7)	129·5% (87·2 to 192·5)	23·9% (3·4 to 55·6)	8·70 (7·60 to 9·90)	161·3 (137·8 to 185·2)	111·5% (78·7 to 156·6)	16·6% (−1·3 to 40·0)	36·3 (32·0 to 42·1)	5415·3 (4763·0 to 6197·4)	108·5% (75·5 to 156·0)	15·0% (−3·4 to 40·5)
		Tanzania	53·4 (45·7 to 63·7)	147·1 (128·4 to 170·4)	147·8% (98·9 to 206·1)	−2·8% (−20·4 to 18·9)	33·1 (28·8 to 38·5)	100·3 (85·6 to 116·8)	132·0% (94·8 to 178·1)	−7·4% (−22·3 to 11·6)	115 (98·1 to 134)	2889·3 (2490·7 to 3300·9)	105·4% (67·7 to 148·4)	−16·0% (−30·4 to 0·8)
		Uganda	61·4 (50·7 to 73·3)	244·1 (208·7 to 284·9)	262·3% (186·2 to 377·0)	36·4% (10·7 to 71·2)	34·1 (29·7 to 38·4)	155·7 (137·2 to 174·7)	197·8% (149·4 to 271·4)	15·5% (−1·5 to 38·2)	148 (126 to 168)	5239·8 (4532·1 to 5944·9)	190·9% (134·8 to 270·2)	17·8% (−1·2 to 46·2)
		Zambia	22·3 (17·9 to 27·1)	211·3 (174·0 to 251·7)	266·4% (184·6 to 374·8)	37·1% (8·8 to 76·1)	12·4 (10·5 to 14·6)	138·0 (118·2 to 162·4)	219·3% (156·8 to 299·6)	23·5% (−0·6 to 56·2)	52·0 (43·8 to 61·6)	4400·0 (3755·7 to 5188·0)	206·5% (145·3 to 284·3)	22·2% (−1·4 to 54·5)
	Southern sub-Saharan Africa		137 (120 to 153)	189·9 (170·0 to 210·2)	195·1% (156·2 to 248·2)	20·7% (6·4 to 40·1)	84·0 (76·3 to 93·6)	123·3 (112·3 to 136·2)	167·5% (133·9 to 208·8)	9·7% (−4·0 to 25·8)	256 (226 to 294)	3413·6 (3033·9 to 3875·8)	166·6% (127·0 to 214·2)	13·0% (−1·9 to 31·9)
		Botswana	2·90 (2·30 to 3·60)	143·3 (120·0 to 171·0)	282·3% (203·0 to 374·4)	45·1% (19·4 to 76·7)	1·50 (1·30 to 1·80)	79·9 (67·2 to 90·5)	240·4% (173·3 to 324·1)	32·1% (7·8 to 63·3)	5·60 (4·50 to 6·70)	2596·2 (2136·3 to 3071·1)	196·4% (130·3 to 277·0)	28·8% (2·6 to 63·1)
		Eswatini	1·90 (1·40 to 2·50)	199·9 (152·9 to 253·9)	76·0% (28·4 to 151·9)	−15·6% (−36·5 to 17·0)	1·00 (0·800 to 1·20)	108·6 (87·7 to 136·0)	61·7% (21·7 to 121·3)	−23·3% (−41·2 to 3·7)	4·00 (2·90 to 5·30)	3989·3 (3038·2 to 5245·5)	49·0% (9·6 to 108·8)	−25·8% (−44·4 to 2·9)
		Lesotho	2·30 (1·90 to 2·90)	170·5 (138·5 to 208·0)	66·5% (23·9 to 122·4)	9·7% (−17·1 to 44·2)	1·60 (1·30 to 2·00)	120·6 (95·6 to 149·4)	62·7% (22·1 to 117·0)	6·5% (−19·0 to 40·7)	5·00 (3·90 to 6·30)	3475·8 (2739·7 to 4394·2)	56·8% (14·5 to 115·9)	7·2% (−22·1 to 48·4)
		Namibia	3·60 (2·90 to 4·30)	161·4 (134·1 to 187·2)	214·8% (161·9 to 287·5)	23·4% (4·3 to 50·4)	1·80 (1·50 to 2·00)	87·0 (74·0 to 97·7)	176·7% (132·0 to 229·8)	10·8% (−5·9 to 31·0)	7·40 (5·90 to 8·50)	3056·7 (2513·1 to 3472·4)	159·3% (118·0 to 212·6)	6·2% (−11·3 to 27·4)
		South Africa	113 (99·3 to 129)	202·2 (179·4 to 226·8)	216·2% (173·8 to 277·2)	22·6% (6·9 to 44·0)	69·4 (62·9 to 77·7)	130·8 (118·9 to 144·8)	183·4% (147·2 to 229·3)	10·5% (−3·7 to 28·2)	207 (181 to 240)	3547·6 (3142·0 to 4030·3)	184·2% (142·1 to 237·0)	13·5% (−2·3 to 33·4)
		Zimbabwe	12·6 (10·8 to 14·7)	130·6 (113·4 to 153·5)	106·8% (71·2 to 148·5)	2·5% (−13·7 to 22·0)	8·70 (7·50 to 10·3)	95·0 (82·5 to 111·8)	105·5% (70·7 to 147·2)	1·4% (−15·6 to 21·2)	27·8 (23·7 to 33·2)	2658·3 (2275·9 to 3177·4)	115·5% (76·5 to 163·7)	9·6% (−10·4 to 33·2)
		Western Sub-saharan Africa	415 (354 to 475)	150·2 (129·2 to 171·1)	216·2% (167·1 to 280·6)	23·7% (5·0 to 47·4)	248 (211 to 280)	101·0 (85·2 to 115·3)	170·2% (131·2 to 213·3)	10·1% (−7·1 to 31·0)	946 (808 to 1080)	3017·4 (2567·4 to 3414·6)	162·2% (121·7 to 205·4)	5·8% (−9·6 to 22·2)
		Benin	8·20 (6·70 to 9·80)	108·0 (88·4 to 127·9)	246·7% (183·7 to 322·2)	17·6% (−4·7 to 44·5)	5·10 (4·20 to 6·00)	74·5 (61·4 to 87·5)	190·9% (142·1 to 245·9)	1·9% (−16·2 to 23·7)	20·1 (16·5 to 23·9)	2246·2 (1871·2 to 2621·8)	192·9% (149·1 to 249·1)	4·9% (−11·2 to 24·4)
		Burkina Faso	14·2 (12·3 to 16·4)	116·3 (100·3 to 133·7)	137·9% (93·6 to 184·7)	−1·6% (−18·9 to 16·7)	9·70 (8·30 to 11·0)	87·5 (74·3 to 101·3)	111·2% (73·1 to 152·9)	−11·0% (−25·8 to 7·7)	32·4 (28·2 to 36·3)	2362·6 (2051·2 to 2665·5)	99·4% (62·5 to 137·3)	−13·1% (−29·1 to 4·1)
		Cameroon	29·4 (24·4 to 34·3)	167·6 (138·6 to 191·8)	265·2% (198·1 to 348·1)	12·7% (−8·3 to 35·3)	18·4 (15·7 to 21·1)	120·5 (100·5 to 139·3)	210·1% (156·6 to 267·5)	0·6% (−18·2 to 20·5)	69·1 (59·7 to 79·3)	3471·8 (2977·4 to 3999·3)	207·3% (155·1 to 265·7)	−3·3% (−20·1 to 14·4)
		Cabo Verde	0·80 (0·70 to 0·90)	179·9 (151·8 to 208·1)	160·0% (107·6 to 211·1)	36·2% (8·8 to 63·8)	0·600 (0·500 to 0·600)	127·7 (110·5 to 146·4)	111·1% (74·7 to 148·4)	13·1% (−6·7 to 34·0)	1·50 (1·30 to 1·80)	3345·0 (2883·8 to 3849·8)	131·7% (93·1 to 175·1)	21·4% (0·3 to 43·7)
		Chad	11·9 (9·70 to 14·9)	146·0 (122·1 to 176·6)	151·0% (101·9 to 211·2)	1·8% (−17·1 to 25·6)	8·10 (6·70 to 9·60)	113·3 (95·6 to 133·6)	123·6% (84·4 to 173·4)	−3·4% (−21·5 to 22·9)	30·9 (25·6 to 37·9)	3245·3 (2692·1 to 3893·6)	143·0% (101·6 to 200·5)	−5·3% (−22·2 to 16·5)
		Côte d’Ivoire	29·1 (24·3 to 35·2)	155·8 (131·6 to 184·6)	389·5% (290·8 to 499·9)	44·9% (18·7 to 73·3)	17·1 (14·4 to 19·9)	106·2 (91·0 to 121·0)	317·0% (243·5 to 401·4)	23·8% (1·9 to 50·5)	69·3 (57·7 to 82·0)	3267·9 (2752·2 to 3781·7)	294·8% (224·6 to 390·7)	29·5% (6·7 to 56·1)
		The Gambia	1·20 (1·00 to 1·50)	84·4 (68·0 to 101·9)	295·4% (207·8 to 386·9)	42·0% (13·2 to 76·2)	0·800 (0·600 to 1·00)	58·8 (47·7 to 71·2)	275·4% (194·0 to 373·9)	33·9% (5·2 to 74·7)	3·10 (2·40 to 3·80)	1878·7 (1496·6 to 2314·5)	229·0% (158·0 to 313·2)	28·8% (0·5 to 62·2)
		Ghana	29·1 (24·7 to 34·1)	136·3 (115·7 to 158·6)	199·1% (142·5 to 273·3)	12·4% (−8·3 to 39·5)	17·8 (15·6 to 20·6)	92·5 (79·9 to 106·7)	166·8% (121·3 to 223·7)	−1·1% (−19·1 to 20·9)	63·8 (55·8 to 73·3)	2751·7 (2409·1 to 3170·6)	134·1% (91·8 to 184·3)	−3·8% (−20·8 to 16·5)
		Guinea	11·4 (9·60 to 13·9)	144·9 (122·9 to 173·2)	141·0% (92·0 to 204·3)	19·5% (−3·9 to 51·5)	7·40 (6·20 to 8·70)	103·8 (86·5 to 122·2)	112·0% (73·1 to 164·7)	9·0% (−12·2 to 37·8)	27·9 (23·4 to 33·0)	3233·7 (2725·5 to 3798·6)	113·0% (74·8 to 169·1)	9·1% (−10·2 to 37·4)
		Guinea-Bissau	1·60 (1·30 to 1·90)	137·6 (114·8 to 163·5)	201·9% (134·9 to 272·5)	44·5% (16·5 to 77·3)	1·00 (0·900 to 1·20)	101·4 (84·9 to 120·0)	163·8% (110·5 to 227·6)	33·4% (8·9 to 64·3)	4·10 (3·40 to 4·80)	3100·0 (2598·5 to 3713·3)	162·6% (104·5 to 232·5)	31·7% (4·3 to 63·4)
		Liberia	6·10 (4·80 to 7·20)	175·5 (141·3 to 205·8)	215·8% (144·7 to 303·4)	30·1% (3·2 to 62·3)	3·70 (3·10 to 4·30)	123·7 (99·7 to 145·0)	154·4% (107·6 to 206·9)	12·0% (−10·1 to 36·1)	14·5 (11·8 to 16·8)	3782·4 (3110·6 to 4359·5)	164·8% (114·1 to 220·6)	12·8% (−8·2 to 37·1)
		Mali	22·4 (19·2 to 25·8)	175·3 (150·3 to 199·8)	218·1% (164·2 to 291·4)	28·1% (8·3 to 55·2)	14·0 (12·1 to 15·8)	122·8 (105·5 to 139·3)	183·1% (143·8 to 233·6)	16·1% (0·2 to 36·6)	55·4 (48·2 to 63·3)	3872·0 (3341·9 to 4353·7)	166·5% (128·4 to 222·2)	13·2% (−2·5 to 34·7)
		Mauritania	5·00 (4·00 to 6·00)	189·5 (152·0 to 225·6)	278·2% (195·9 to 382·9)	62·3% (27·3 to 107·4)	3·20 (2·50 to 3·80)	130·8 (103·8 to 157·1)	208·7% (147·0 to 294·9)	36·3% (7·1 to 75·6)	10·5 (8·30 to 12·6)	3690·8 (2914·3 to 4424·2)	197·4% (136·5 to 277·2)	33·9% (6·8 to 70·8)
		Niger	14·0 (11·5 to 17·9)	102·5 (85·8 to 127·6)	288·2% (219·5 to 402·8)	23·3% (2·3 to 51·5)	8·50 (7·00 to 10·8)	71·8 (60·2 to 89·0)	250·3% (191·4 to 335·6)	13·3% (−6·9 to 40·2)	41·1 (33·5 to 53·2)	2315·2 (1937·1 to 2959·0)	241·4% (179·7 to 340·3)	13·0% (−6·2 to 39·6)
		Nigeria	202 (167 to 236)	158·9 (131·0 to 187·3)	207·7% (152·1 to 283·1)	27·9% (6·0 to 58·9)	115 (94·5 to 136)	101·9 (83·3 to 122·9)	157·7% (111·9 to 212·9)	13·5% (−7·9 to 40·3)	438 (365 to 517)	3022·9 (2484·9 to 3603·3)	150·4% (106·5 to 198·9)	5·2% (−13·5 to 26·7)
		São Tomé and Príncipe	0·200 (0·200 to 0·300)	167·4 (140·4 to 196·7)	186·6% (120·7 to 253·5)	42·2% (11·5 to 72·1)	0·200 (0·100 to 0·200)	115·0 (99·3 to 133·5)	139·5% (90·8 to 183·3)	27·1% (2·1 to 50·0)	0·500 (0·400 to 0·600)	3225·6 (2751·2 to 3738·6)	144·4% (91·0 to 197·7)	24·0% (−1·9 to 48·0)
		Senegal	13·6 (11·7 to 15·7)	126·2 (108·1 to 145·4)	254·3% (186·3 to 331·5)	37·3% (13·3 to 65·1)	8·30 (7·00 to 9·60)	84·0 (70·6 to 97·7)	222·5% (167·9 to 286·6)	26·3% (5·7 to 52·3)	29·9 (25·6 to 34·4)	2505·4 (2133·4 to 2914·7)	178·7% (130·1 to 240·0)	18·4% (−1·3 to 43·3)
		Sierra Leone	7·00 (5·90 to 8·40)	150·3 (128·6 to 176·8)	125·4% (75·0 to 192·2)	20·1% (−6·6 to 52·7)	4·50 (3·80 to 5·20)	108·4 (91·4 to 125·6)	96·4% (55·5 to 144·2)	10·3% (−12·9 to 41·1)	16·9 (14·3 to 19·8)	3245·7 (2749·1 to 3745·0)	103·8% (62·6 to 152·8)	8·1% (−13·9 to 34·9)
		Togo	7·30 (6·00 to 8·70)	135·0 (112·0 to 158·5)	290·5% (202·9 to 373·4)	19·1% (−4·5 to 43·3)	4·50 (3·80 to 5·40)	93·4 (76·4 to 110·2)	247·0% (178·8 to 316·3)	4·7% (−14·3 to 27·2)	16·8 (13·9 to 20·0)	2834·3 (2358·2 to 3370·3)	216·8% (155·4 to 283·7)	7·1% (−14·1 to 28·4)

Data in parentheses are 95% uncertainty intervals. DALY=disability-adjusted life-year. GBD=Global Burden of Diseases, Injuries, and Risk Factors Study.

* Includes countries from north Africa only from the GBD super-region north Africa and the Middle East.

† From the GBD super-region southeast Asia.

Cancer incidence by region, country, and sex presented in table 1, figure 1, and the appendix (pp 104–115). In 2023, Nigeria, Ethiopia, Egypt, and South Africa recorded the highest absolute numbers of new cancer cases. The highest age-standardised incidence rates were observed in Equatorial Guinea, Ethiopia, and Uganda. The lowest age-standardised incidence rates were reported in Somalia, The Gambia, and Comoros across both sexes and all ages (table 1; appendix pp 103–113).

**Figure 1:**
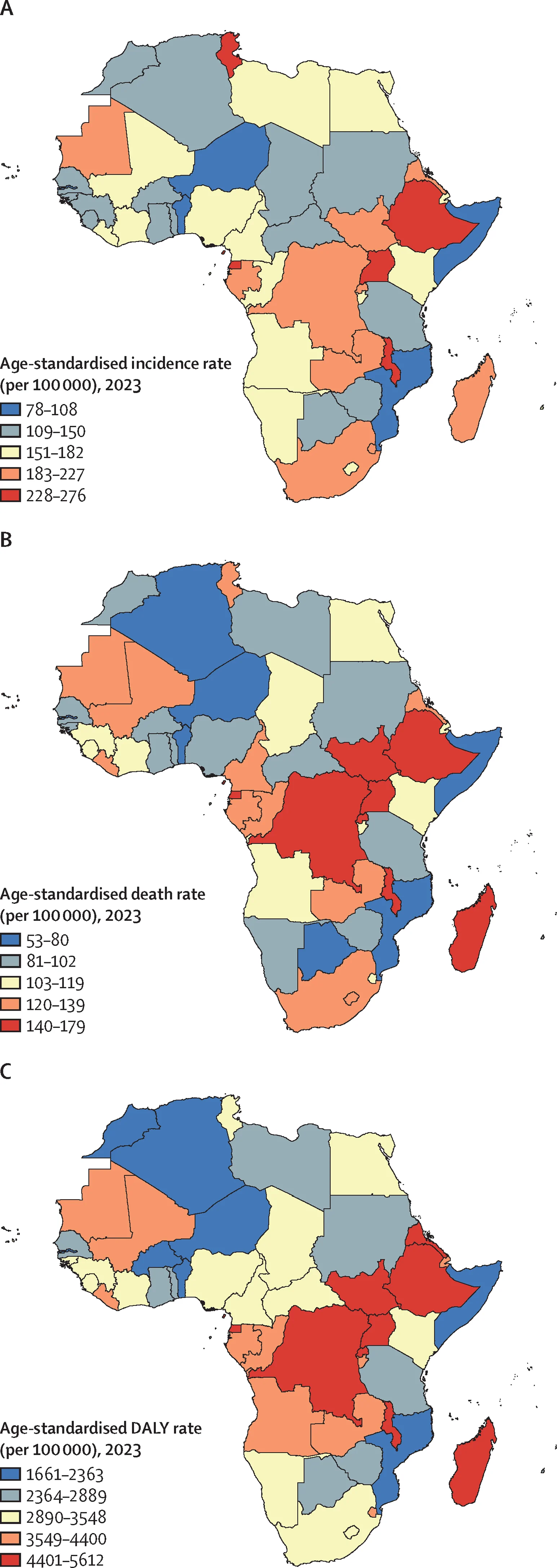
Age-standardised incidence (A), death (B), and DALY (C) rate per 100 000 of cancer in Africa, 2023 DALY=disability-adjusted life-year.

In 2010, cervical, breast, liver, non-melanoma skin, prostate, and colorectal cancers were the most frequently observed in all ages and both sexes (GBD results tool). In 2023, breast cancer (267 000 [95% UI 197 000–349 000]), cervical cancer (253 000 [178 000–361 000]), prostate cancer (97 000 [67 000–132 000]), liver cancer (94 000 [64 000–132 000]), and colorectal cancer (89 000 [68 000–112 000]) had the highest incidence in both sexes and all ages. Between 2010 and 2023, age-standardised increases were highest in breast cancer (72·7% [24·0–145·3]), ovarian cancer (72·2% [20·5–158·0]), thyroid cancer (71·3% [9·4–145·7]), and melanoma (59·8% [3·5–135·4]). In 2023, leading female cancers were breast, cervical, colorectal, and ovarian, with breast and cervical together accounting for 53·5% (44·7–62·2%) of all female cancers. In males, prostate, liver, tracheal, bronchus and lung, and colorectal cancers were the leading types, together accounting for 44·8% (36·2–54·3) of all male cancers in Africa in 2023 (table 2; appendix pp 116–127).

**Table 2:** Cancer incidence, death, and DALY counts and age-standardised rates in 2023 and percentage change in counts and age-standardised rates between 1990 and 2023 in Africa for all ages and sexes combined by cancer type

		**Incidence**				**Deaths**				**DALYs**			
		Cases, thousands, 2023	Age-standardised rate, 2023	Percentage change in cases, 1990–2023	Percentage change in age-standardised rate, 1990–2023	Count, thousands, 2023	Age-standardised rate, 2023	Percentage change in count, 1990–2023	Percentage change in age-standardised rate, 1990–2023	Count, ten thousands, 2023	Age-standardised rate, 2023	Percentage change in count, 1990–2023	Percentage change in age-standardised rate, 1990–2023
Total cancers		1540 (1370 to 1750)	172·1 (154·1 to 193·4)	229·0% (183·5 to 286·2)	31·1% (13·6 to 51·3)	923 (824 to 1020)	114·2 (100·6 to 125·8)	185·0% (151·9 to 219·4)	15·5% (2·3 to 31·9)	3390 (3060 to 3710)	3443·1 (3092·9 to 3781·0)	162·5% (128·5 to 194·9)	11·5% (−1·6 to 24·6)
Oesophageal cancer		44·0 (34·3 to 57·2)	5·7 (4·5 to 7·4)	163·4% (88·4 to 267·3)	2·1% (−26·9 to 42·1)	42·3 (33·0 to 54·9)	5·7 (4·5 to 7·4)	162·5% (86·7 to 266·3)	1·6% (−27·1 to 41·7)	126 (98·8 to 166)	152·1 (118·7 to 198·1)	158·7% (84·2 to 262·8)	0·0% (−29·0 to 39·7)
Stomach cancer		58·6 (42·4 to 71·8)	7·4 (5·3 to 9·1)	124·0% (53·6 to 222·6)	−13·0% (−40·9 to 24·6)	54·3 (39·2 to 66·5)	7·3 (5·2 to 8·9)	118·4% (50·9 to 216·2)	−15·5% (−42·2 to 20·5)	171 (125 to 209)	195·5 (141·7 to 239·1)	115·1% (50·6 to 212·4)	−16·4% (−41·8 to 21·3)
Liver cancer		94·0 (64·0 to 132)	11·2 (7·7 to 15·6)	106·6% (30·1 to 218·8)	−18·2% (−48·2 to 26·6)	87·0 (59·0 to 122)	10·9 (7·5 to 15·1)	103·7% (27·4 to 216·6)	−19·2% (−49·3 to 25·1)	302 (204 to 429)	320·8 (218·6 to 452·8)	94·7% (20·5 to 207·1)	−21·1% (−50·7 to 22·4)
	Liver cancer due to hepatitis B	34·6 (22·1 to 49·4)	3·8 (2·4 to 5·4)	85·8% (11·7 to 203·9)	−28·8% (−57·6 to 17·5)	31·0 (19·8 to 44·6)	3·5 (2·2 to 5·1)	82·9% (7·9 to 201·8)	−29·6% (−58·5 to 16·9)	119 (75·0 to 170)	119·0 (75·8 to 171·1)	82·8% (8·6 to 204·9)	−30·0% (−58·5 to 15·8)
	Liver cancer due to hepatitis C	26·2 (17·2 to 38·1)	3·6 (2·4 to 5·3)	120·0% (49·4 to 232·8)	−15·0% (−42·9 to 29·6)	25·6 (17·0 to 37·4)	3·6 (2·4 to 5·3)	116·2% (47·7 to 228·0)	−17·0% (−44·4 to 27·5)	71·2 (46·4 to 104)	89·5 (59·0 to 130·9)	115·8% (47·6 to 226·5)	−16·8% (−43·2 to 26·4)
	Liver cancer due to alcohol use	15·4 (9·20 to 23·9)	1·9 (1·2 to 3·0)	119·4% (28·8 to 258·2)	−14·3% (−49·7 to 39·8)	14·6 (8·90 to 22·4)	1·9 (1·2 to 2·9)	117·7% (27·6 to 253·1)	−14·7% (−50·0 to 38·1)	45·6 (27·1 to 71·0)	53·2 (32·0 to 82·2)	116·3% (27·9 to 252·4)	−16·5% (−50·9 to 35·5)
	Liver cancer due to other causes	5·30 (3·40 to 8·00)	0·6 (0·4 to 0·9)	129·2% (52·2 to 260·0)	−9·7% (−39·7 to 41·0)	4·70 (2·90 to 7·10)	0·5 (0·3 to 0·8)	124·2% (49·0 to 255·8)	−11·4% (−40·8 to 39·5)	18·2 (11·4 to 27·4)	17·9 (11·1 to 27·1)	116·1% (41·9 to 245·2)	−13·4% (−42·5 to 36·6)
	Liver cancer due to NASH	9·10 (5·90 to 13·2)	1·2 (0·7 to 1·7)	182·5% (82·4 to 331·7)	11·3% (−27·7 to 70·2)	8·70 (5·60 to 12·4)	1·2 (0·7 to 1·7)	179·4% (81·4 to 325·5)	9·8% (−27·9 to 67·5)	26·8 (17·1 to 38·6)	30·9 (19·8 to 44·7)	168·9% (74·6 to 313·0)	7·1% (−30·4 to 63·2)
	Hepatoblastoma	3·10 (1·80 to 4·90)	0·2 (0·1 to 0·2)	36·2% (−22·8 to 156·0)	−27·8% (−59·0 to 35·2)	2·40 (1·40 to 3·90)	0·1 (0·1 to 0·2)	21·0% (−33·2 to 129·4)	−35·7% (−64·4 to 21·3)	21·3 (12·3 to 34·1)	10·4 (6·0 to 16·6)	21·2% (−33·1 to 130·1)	−35·5% (−64·3 to 22·0)
Larynx cancer		12·9 (9·40 to 18·0)	1·7 (1·2 to 2·3)	208·7% (99·4 to 331·3)	20·3% (−22·2 to 69·7)	9·30 (7·00 to 12·9)	1·3 (0·9 to 1·7)	175·1% (79·0 to 281·7)	7·4% (−29·4 to 50·0)	28·4 (21·4 to 38·5)	34·2 (25·7 to 46·8)	171·9% (76·2 to 276·2)	5·7% (−31·7 to 46·3)
Tracheal, bronchus, and lung cancer		74·0 (57·7 to 94·2)	10·0 (7·8 to 12·7)	234·5% (154·4 to 376·8)	32·3% (0·0 to 89·3)	72·0 (56·0 to 92·0)	10·0 (7·8 to 12·9)	230·7% (151·8 to 374·3)	30·6% (−0·9 to 88·3)	203 (159 to 256)	253·4 (198·5 to 323·2)	222·1% (146·7 to 359·8)	26·9% (−3·0 to 81·5)
Breast cancer		267 (197 to 349)	28·2 (20·9 to 36·8)	544·1% (299·6 to 829·7)	140·0% (51·4 to 244·9)	129 (94·0 to 166)	14·8 (10·9 to 19·1)	402·7% (213·1 to 617·4)	87·3% (17·6 to 166·2)	505 (367 to 651)	509·2 (372·0 to 657·0)	407·9% (212·5 to 623·3)	91·8% (19·5 to 172·4)
Cervical cancer		253 (178 to 361)	23·5 (16·6 to 32·9)	254·4% (130·2 to 498·0)	35·2% (−11·4 to 126·7)	107 (75·0 to 151)	11·3 (7·8 to 15·7)	205·4% (93·2 to 408·2)	17·2% (−25·1 to 95·0)	457 (319 to 647)	430·7 (302·9 to 602·7)	200·5% (89·1 to 402·3)	16·9% (−25·7 to 94·7)
Uterine cancer		23·3 (15·9 to 34·7)	2·8 (1·9 to 4·1)	382·2% (164·0 to 724·6)	83·0% (2·1 to 216·5)	8·50 (6·00 to 12·6)	1·1 (0·8 to 1·7)	259·3% (102·3 to 490·8)	37·7% (−22·1 to 128·8)	27·4 (19·2 to 40·2)	31·7 (22·4 to 46·9)	265·4% (103·8 to 492·2)	40·7% (−21·0 to 130·4)
Prostate cancer		97·0 (67·0 to 132)	15·1 (10·5 to 20·7)	300·1% (171·6 to 542·8)	52·7% (4·5 to 143·2)	66·8 (44·7 to 94·9)	11·4 (7·6 to 16·2)	222·7% (118·9 to 405·0)	24·5% (−15·9 to 96·6)	138 (94·3 to 195)	214·0 (145·1 to 302·1)	216·5% (114·6 to 389·8)	24·6% (−15·2 to 92·4)
Colon and rectum cancer		89·0 (68·0 to 112)	11·7 (8·9 to 14·7)	323·6% (223·2 to 486·1)	64·6% (26·0 to 126·4)	68·0 (52·0 to 86·0)	9·4 (7·3 to 12·0)	273·7% (183·9 to 418·1)	44·8% (10·0 to 100·2)	204 (158 to 256)	241·0 (187·4 to 303·7)	262·7% (173·9 to 399·9)	42·4% (8·2 to 96·8)
Lip and oral cavity cancer		21·9 (17·1 to 27·5)	2·6 (2·0 to 3·3)	218·3% (119·8 to 355·3)	24·2% (−14·5 to 79·3)	13·4 (10·5 to 17·0)	1·7 (1·4 to 2·2)	185·9% (98·9 to 308·6)	11·6% (−22·6 to 58·6)	45·3 (35·6 to 57·1)	49·8 (39·1 to 63·1)	177·2% (94·3 to 288·7)	8·6% (−24·3 to 54·2)
Nasopharynx cancer		12·9 (9·20 to 17·2)	1·3 (0·9 to 1·7)	175·8% (60·0 to 314·5)	10·1% (−34·9 to 62·8)	8·90 (6·50 to 11·7)	0·9 (0·7 to 1·2)	144·8% (46·1 to 267·4)	−2·3% (−41·2 to 46·8)	38·0 (27·4 to 50·0)	35·3 (25·5 to 46·3)	131·4% (37·7 to 246·9)	−5·7% (−43·6 to 40·9)
Other pharynx cancer		4·20 (3·00 to 5·70)	0·5 (0·3 to 0·7)	263·1% (144·7 to 445·0)	40·1% (−6·7 to 110·7)	3·10 (2·20 to 4·20)	0·4 (0·3 to 0·5)	226·8% (121·7 to 392·9)	26·8% (−14·0 to 90·2)	10·5 (7·60 to 14·1)	11·7 (8·4 to 15·8)	222·4% (118·2 to 386·3)	24·5% (−15·6 to 88·3)
Gallbladder and biliary tract cancer		7·10 (5·30 to 9·30)	1·0 (0·7 to 1·3)	245·3% (146·6 to 354·4)	35·4% (−3·4 to 79·6)	6·80 (5·10 to 8·90)	1·0 (0·7 to 1·3)	235·8% (140·6 to 342·7)	31·1% (−6·4 to 74·4)	18·7 (13·7 to 24·1)	23·7 (17·5 to 30·7)	228·2% (133·7 to 327·5)	28·4% (−8·2 to 68·6)
Pancreatic cancer		22·6 (18·0 to 27·7)	3·1 (2·4 to 3·8)	355·1% (248·3 to 528·6)	78·1% (35·8 to 145·3)	21·9 (17·5 to 26·8)	3·1 (2·5 to 3·8)	351·2% (245·8 to 521·1)	75·6% (33·6 to 140·9)	61·5 (48·8 to 75·4)	76·7 (61·2 to 94·2)	340·2% (237·0 to 508·1)	72·7% (32·3 to 137·9)
Ovarian cancer		40·9 (28·6 to 56·2)	4·2 (3·0 to 5·7)	455·1% (224·7 to 771·6)	114·8% (28·7 to 233·7)	24·1 (17·1 to 32·7)	2·8 (2·0 to 3·8)	415·1% (206·0 to 713·9)	98·7% (19·4 to 216·5)	91·9 (64·9 to 123)	93·2 (66·2 to 125·4)	407·5% (198·7 to 697·5)	99·3% (18·4 to 214·0)
Testicular cancer		4·80 (2·60 to 8·00)	0·4 (0·2 to 0·6)	282·7% (56·8 to 731·7)	54·6% (−35·3 to 232·7)	1·30 (0·800 to 2·00)	0·1 (0·1 to 0·2)	136·5% (5·3 to 342·3)	−2·3% (−55·0 to 79·4)	7·30 (4·30 to 11·3)	5·5 (3·3 to 8·5)	128·3% (−0·2 to 333·3)	−5·5% (−58·1 to 76·1)
Kidney cancer		15·1 (10·7 to 20·6)	1·5 (1·0 to 2·0)	226·2% (111·2 to 372·3)	43·5% (−2·6 to 104·4)	7·30 (5·00 to 9·70)	0·9 (0·6 to 1·2)	185·9% (97·9 to 307·4)	22·0% (−14·3 to 75·3)	29·8 (20·3 to 39·4)	27·4 (18·7 to 36·4)	156·6% (73·1 to 261·0)	16·6% (−19·3 to 65·7)
Bladder cancer		31·5 (23·1 to 42·6)	4·4 (3·2 to 6·0)	158·5% (74·1 to 301·3)	5·2% (−28·7 to 60·0)	16·5 (12·1 to 23·3)	2·6 (1·9 to 3·6)	144·4% (72·3 to 254·4)	−2·5% (−30·9 to 39·5)	41·6 (30·6 to 58·1)	56·1 (41·5 to 78·8)	125·6% (56·4 to 237·0)	−8·5% (−36·0 to 35·1)
Brain and CNS cancer		25·5 (18·6 to 34·5)	2·2 (1·6 to 2·9)	143·3% (79·8 to 231·4)	12·9% (−13·3 to 54·7)	17·8 (13·2 to 22·8)	1·7 (1·3 to 2·2)	139·7% (80·9 to 223·0)	10·9% (−13·4 to 52·2)	92·3 (69·0 to 119)	69·5 (51·4 to 89·0)	110·8% (56·6 to 181·7)	−0·2% (−24·4 to 34·2)
Thyroid cancer		40·0 (25·9 to 57·5)	3·5 (2·3 to 4·9)	437·0% (220·3 to 873·2)	115·4% (31·3 to 278·2)	6·50 (4·80 to 8·80)	0·7 (0·5 to 1·0)	229·3% (126·5 to 405·0)	35·9% (−5·6 to 110·8)	28·3 (20·6 to 39·4)	26·1 (19·1 to 35·8)	210·7% (104·8 to 369·3)	31·3% (−10·8 to 99·5)
Mesothelioma		1·10 (0·800 to 1·40)	0·1 (0·1 to 0·2)	273·7% (155·2 to 459·9)	47·0% (−1·8 to 122·4)	1·00 (0·700 to 1·40)	0·1 (0·1 to 0·2)	269·9% (149·9 to 455·9)	45·0% (−4·0 to 119·0)	3·30 (2·20 to 4·30)	3·7 (2·6 to 4·9)	260·6% (149·4 to 439·7)	42·2% (−2·9 to 112·8)
Hodgkin lymphoma		12·4 (8·00 to 17·0)	0·9 (0·6 to 1·3)	88·3% (23·3 to 194·7)	−23·0% (−49·3 to 19·6)	7·40 (4·70 to 10·3)	0·6 (0·4 to 0·8)	52·3% (−2·3 to 140·5)	−36·5% (−58·9 to −0·4)	42·4 (26·9 to 59·0)	29·8 (19·1 to 41·4)	47·7% (−7·2 to 132·6)	−37·5% (−59·8 to −1·7)
Non-Hodgkin lymphoma		57·9 (41·9 to 81·4)	5·3 (3·9 to 7·3)	186·9% (76·4 to 348·6)	22·1% (−23·9 to 86·0)	36·9 (27·3 to 48·8)	3·9 (2·8 to 5·1)	144·1% (59·1 to 265·3)	5·0% (−31·9 to 57·7)	175 (127 to 235)	141·5 (105·1 to 187·6)	123·1% (46·0 to 234·0)	−0·8% (−34·9 to 48·6)
	Burkitt lymphoma	5·40 (3·00 to 9·90)	0·3 (0·2 to 0·6)	71·8% (−17·9 to 288·6)	−15·7% (−58·9 to 92·6)	2·90 (1·80 to 5·10)	0·2 (0·1 to 0·3)	46·5% (−24·0 to 197·3)	−26·9% (−61·8 to 50·1)	21·2 (12·2 to 37·8)	12·0 (7·1 to 21·0)	37·0% (−28·9 to 179·2)	−33·2% (−65·3 to 35·7)
	Other non-Hodgkin lymphoma	52·5 (37·3 to 72·4)	5·0 (3·6 to 6·8)	208·1% (92·9 to 368·6)	25·6% (−21·3 to 88·7)	34·0 (25·1 to 44·5)	3·7 (2·7 to 4·8)	159·0% (70·0 to 290·4)	7·3% (−29·8 to 60·8)	154 (112 to 206)	129·5 (95·8 to 170·0)	144·3% (57·9 to 271·8)	3·9% (−31·8 to 56·6)
Multiple myeloma		10·1 (7·20 to 13·8)	1·3 (0·9 to 1·8)	382·8% (212·7 to 595·1)	85·7% (21·1 to 168·5)	8·70 (6·40 to 11·8)	1·2 (0·9 to 1·6)	348·5% (210·3 to 552·9)	73·3% (20·9 to 155·8)	25·6 (19·0 to 34·4)	31·1 (23·0 to 42·0)	349·4% (204·6 to 544·2)	73·6% (19·1 to 151·0)
Leukaemia		50·2 (39·0 to 64·3)	4·2 (3·2 to 5·3)	92·5% (40·0 to 174·9)	−10·6% (−34·7 to 25·6)	37·6 (29·3 to 48·9)	3·4 (2·7 to 4·4)	83·7% (28·0 to 162·3)	−14·6% (−40·0 to 22·3)	2100 (163 to 278)	147·2 (114·9 to 191·9)	65·3% (14·0 to 137·4)	−22·5% (−45·9 to 10·0)
	Acute lymphoid leukaemia	24·3 (16·8 to 35·3)	1·5 (1·0 to 2·2)	70·7% (14·6 to 155·1)	−19·8% (−45·9 to 18·3)	16·9 (11·5 to 24·1)	1·1 (0·7 to 1·5)	59·7% (6·0 to 133·2)	−24·7% (−50·1 to 10·1)	120 (83·3 to 170)	69·0 (47·1 to 98·8)	52·5% (0·5 to 126·8)	−27·7% (−51·9 to 5·2)
	Chronic lymphoid leukaemia	3·00 (2·10 to 4·20)	0·4 (0·3 to 0·6)	172·3% (61·1 to 382·8)	7·6% (−36·2 to 87·7)	2·10 (1·40 to 2·80)	0·3 (0·2 to 0·4)	122·6% (33·1 to 277·3)	−8·9% (−44·7 to 53·7)	5·30 (3·50 to 7·30)	7·0 (4·7 to 9·7)	107·3% (21·5 to 254·2)	−16·5% (−50·6 to 41·9)
	Acute myeloid leukaemia	10·7 (7·00 to 15·6)	1·0 (0·6 to 1·3)	106·8% (32·1 to 243·3)	−6·4% (−39·1 to 50·3)	9·00 (5·90 to 13·1)	0·9 (0·6 to 1·2)	107·2% (35·9 to 240·7)	−6·4% (−38·7 to 52·0)	46·2 (28·9 to 68·9)	35·2 (23·2 to 51·3)	85·0% (19·1 to 209·5)	−15·3% (−44·7 to 38·4)
	Chronic myeloid leukaemia	2·40 (1·40 to 3·80)	0·3 (0·2 to 0·4)	47·3% (−24·2 to 209·5)	−39·1% (−67·8 to 24·2)	2·00 (1·20 to 3·00)	0·2 (0·1 to 0·3)	37·7% (−27·5 to 183·7)	−43·2% (−69·9 to 14·2)	8·30 (4·90 to 13·0)	7·5 (4·5 to 11·5)	28·2% (−34·4 to 175·3)	−46·3% (−71·7 to 10·7)
	Other leukaemia	9·70 (6·30 to 13·9)	1·1 (0·7 to 1·5)	148·8% (49·8 to 343·5)	7·2% (−32·9 to 89·2)	7·60 (4·90 to 10·9)	0·9 (0·6 to 1·3)	141·2% (50·4 to 327·3)	3·8% (−34·8 to 81·9)	29·8 (18·3 to 43·3)	28·5 (17·9 to 40·8)	111·7% (25·4 to 285·2)	−6·7% (−42·0 to 65·0)
Malignant skin melanoma		13·6 (8·70 to 21·7)	1·3 (0·9 to 2·0)	366·5% (190·0 to 584·5)	80·2% (14·2 to 161·0)	6·00 (4·00 to 8·60)	0·7 (0·4 to 0·9)	271·8% (151·1 to 414·6)	43·0% (−2·1 to 98·6)	24·9 (16·3 to 36·5)	23·6 (15·6 to 33·7)	266·6% (141·6 to 416·2)	43·9% (−3·6 to 99·5)
Non-melanoma skin cancer		78·7 (66·1 to 93·6)	10·6 (8·9 to 12·5)	158·5% (154·6 to 163·1)	−1·1% (−2·7 to 0·8)	2·90 (1·60 to 4·40)	0·5 (0·3 to 0·7)	376·5% (212·4 to 639·9)	79·5% (17·4 to 178·2)	8·00 (4·40 to 11·9)	9·9 (5·6 to 14·7)	337·0% (191·2 to 578·7)	72·9% (14·9 to 169·0)
	Non-melanoma skin cancer (squamous-cell carcinoma)	27·7 (23·0 to 34·3)	4·1 (3·3 to 5·0)	164·2% (153·5 to 175·7)	0·3% (−3·5 to 4·8)	2·90 (1·60 to 4·40)	0·5 (0·3 to 0·7)	376·5% (212·4 to 639·9)	79·5% (17·4 to 178·2)	8·00 (4·40 to 11·9)	9·9 (5·6 to 14·7)	337·1% (191·2 to 578·9)	73·0% (14·9 to 169·1)
	Non-melanoma skin cancer (basal-cell carcinoma)	51·0 (40·5 to 64·2)	6·5 (5·2 to 8·1)	155·5% (152·3 to 158·8)	−1·9% (−3·1 to −0·8)	0 (0 to 0)	0·0 (0·0 to 0·0)	0·0% (0·0 to 0·0)	0·0% (0·0 to 0·0)	0 (0 to 0)	0·0 (0·0 to 0·0)	0·0% (0·0 to 0·0)	0·0% (0·0 to 0·0)
Eye cancer		6·90 (4·20 to 11·0)	0·5 (0·4 to 0·9)	51·3% (−31·0 to 198·6)	−32·2% (−62·3 to 6·6)	4·70 (2·70 to 7·60)	0·4 (0·2 to 0·6)	26·8% (−50·7 to 204·5)	−40·5% (−68·6 to 9·0)	29·4 (14·1 to 52·9)	18·9 (10·5 to 31·3)	17·2% (−62·3 to 241·3)	−42·0% (−76·6 to 36·3)
	Retinoblastoma	2·60 (0·900 to 5·90)	0·1 (0·0 to 0·3)	17·0% (−73·5 to 504·2)	−39·4% (−86·3 to 215·6)	2·30 (0·80 to 5·10)	0·1 (0·0 to 0·3)	3·5% (−76·2 to 425·8)	−46·4% (−87·7 to 172·4)	20·0 (7·10 to 44·8)	9·8 (3·5 to 21·9)	3·7% (−76·2 to 426·1)	−46·2% (−87·6 to 172·9)
	Other eye cancers	4·20 (2·70 to 6·80)	0·4 (0·3 to 0·7)	85·2% (19·7 to 193·8)	−29·7% (−52·5 to 8·1)	2·40 (1·50 to 3·80)	0·3 (0·2 to 0·4)	61·8% (0·1 to 141·8)	−37·8% (−61·1 to −6·2)	9·40 (5·90 to 15·0)	9·1 (5·7 to 14·4)	61·9% (0·6 to 140·3)	−36·7% (−60·5 to −6·1)
Soft tissue and other extraosseous sarcomas		19·8 (12·6 to 30·7)	1·6 (1·0 to 2·5)	112·3% (11·9 to 241·8)	−6·5% (−49·8 to 48·4)	11·2 (7·30 to 16·3)	1·0 (0·7 to 1·5)	93·3% (8·3 to 193·1)	−15·2% (−51·8 to 28·0)	61·9 (39·7 to 90·4)	44·6 (29·1 to 65·4)	79·4% (−0·5 to 176·5)	−19·3% (−54·6 to 21·4)
Malignant neoplasm of bone and articular cartilage		18·4 (10·1 to 30·5)	1·4 (0·8 to 2·1)	112·8% (−3·2 to 323·5)	−7·8% (−57·0 to 75·5)	12·5 (7·10 to 19·1)	1·0 (0·6 to 1·5)	96·1% (0·9 to 229·5)	−13·9% (−55·6 to 46·5)	73·2 (40·2 to 113)	49·5 (27·9 to 75·4)	85·2% (−5·1 to 213·3)	−18·7% (−58·3 to 36·3)
Neuroblastoma and other peripheral nervous cell tumours		2·40 (1·40 to 3·90)	0·1 (0·1 to 0·2)	185·2% (55·9 to 416·8)	44·2% (−18·4 to 156·7)	1·10 (0·700 to 1·70)	0·1 (0·0 to 0·1)	138·4% (47·0 to 265·5)	21·4% (−22·9 to 86·0)	8·30 (5·20 to 12·8)	4·6 (3·0 to 7·0)	125·3% (36·5 to 250·9)	14·9% (−29·7 to 76·4)
Other malignant neoplasms		30·2 (22·3 to 38·1)	2·8 (2·1 to 3·6)	91·1% (21·9 to 182·1)	−12·8% (−43·7 to 27·0)	21·1 (15·5 to 26·7)	2·2 (1·6 to 2·8)	80·2% (16·2 to 165·7)	−19·1% (−47·9 to 17·2)	104 (76·4 to 131)	81·0 (59·6 to 102·5)	60·3% (3·0 to 135·9)	−25·3% (−51·6 to 10·4)

Data in parentheses are 95% uncertainty intervals. DALY=disability-adjusted life-year.

Childhood cancer incidence in Africa (ages 0–19 years) increased from 57 400 (95% UI 46 900–70 300) cases in 1990 to 71 400 (60 700–85 700) in 2010, and 104 000 (84 600–130 000) in 2023. In 2023, Africa contributed 27·7% (26·6–29·4) of 377 000 (288 000–489 000) incident childhood cancer cases globally in 2023, and age-specific incidence declined from 16·5 (13·5–20·2) per 100 000 in 1990 to 14·3 (11·6–17·8) in 2023 for both sexes (table 3; GBD results tool).

**Table 3:** Childhood cancer incidence, death, and DALY counts and age-standardised rates in 2023 and percentage change in counts and age-standardised rates between 1990 and 2023 for all ages and sexes combined in Africa

		**Incidence**			**Deaths**				**DALYs**				
		Cases, 2023	Age-standardised rate, 2023	Percentage change in cases, 1990–2023	Percentagechange in age-standardised rate, 1990–2023	Count, 2023	Age-standardised rate, 2023	Percentage change in count, 1990–2023	Percentagechange in age-standardised rate, 1990–2023	Count, 2023	Age-standardisedrate, 2023	Percentagechange in count, 1990–2023	Percentage change in age-standardised rate, 1990–2023
Total cancers		104 000 (84 600to 130 000)	14·3 (11·6 to 17·8)	81·6% (44·9 to 150·9)	−13·5% (−31·0 to 19·5)	54 700 (47 500 to 64 500)	7·5 (6·5 to 8·8)	50·2% (22·3 to 83·5)	−28·5% (−41·8 to −12·6)	4 500 000 (3 900 000 to 5 300 000)	612·4 (533·3 to 723·4)	48·9% (21·1 to 83·3)	−29·1% (−42·4 to −12·7)
Stomach cancer		220 (150to 290)	0·0 (0·0 to 0·0)	73·7% (10·2 to 164·3)	−17·3% (−47·6 to 25·8)	110 (80·0 to 140)	0·0 (0·0 to 0·0)	24·7% (−19·7 to 92·2)	−40·6% (−61·8 to −8·5)	7900 (5600 to 10 300)	1·1 (0·8 to 1·4)	25·1% (−19·5 to 92·8)	−40·4% (−61·7 to −8·2)
Liver cancer		4400 (2680to 6750)	0·6 (0·4 to 0·9)	47·2% (−14·9 to 161·4)	−29·9% (−59·5 to 24·5)	3250 (1920 to 5030)	0·4 (0·3 to 0·7)	27·5% (−25·4 to 135·6)	−39·3% (−64·5 to 12·1)	274 000 (162 000 to 427 000)	37·5 (22·2 to 58·4)	26·9% (−26·3 to 135·5)	−39·6% (−64·9 to 12·1)
	Liver cancer due to hepatitis B	740 (410to 1130)	0·1 (0·1 to 0·1)	74·6% (1·2 to 183·5)	−16·9% (−51·8 to 35·0)	490 (290 to 760)	0·1 (0·0 to 0·1)	47·1% (−10·5 to 153·9)	−30·0% (−57·4 to 20·9)	37 100 (21 600 to 57 400)	5·1 (3·0 to 7·9)	47·4% (−10·4 to 155·4)	−29·8% (−57·3 to 21·6)
	Liver cancer due to other causes	320 (180to 540)	0·0 (0·0 to 0·1)	89·3% (9·2 to 260·4)	−9·9% (−48·0 to 71·6)	200 (110 to 330)	0·0 (0·0 to 0·1)	54·0% (−3·3 to 183·7)	−26·7% (−54·0 to 35·1)	14 900 (8300 to 24 900)	2·0 (1·1 to 3·4)	54·3% (−3·2 to 183·7)	−26·6% (−53·9 to 35·1)
	Liver cancer due to NASH	160 (90·0to 260)	0·0 (0·0 to 0·0)	129·9% (35·8 to 305·8)	9·4% (−35·3 to 93·2)	90·0 (50·0 to 150)	0·0 (0·0 to 0·0)	77·2% (11·7 to 205·4)	−15·7% (−46·8 to 45·4)	6900 (3900 to 11 200)	0·9 (0·5 to 1·5)	77·5% (11·8 to 206·0)	−15·5% (−46·8 to 45·7)
	Hepatoblastoma	3130 (1840to 4920)	0·4 (0·2 to 0·7)	36·2% (−22·8 to 156·0)	−35·1% (−63·3 to 21·9)	2430 (1400 to 3890)	0·3 (0·2 to 0·5)	21·0% (−33·2 to 129·4)	−42·4% (−68·2 to 9·2)	213 000 (123 000 to 341 000)	29·2 (16·8 to 46·7)	21·2% (−33·1 to 130·1)	−42·3% (−68·2 to 9·5)
Tracheal, bronchus, and lung cancer		210 (150to 290)	0·0 (0·0 to 0·0)	140·6% (59·6 to 300·1)	14·6% (−24·0 to 90·5)	200 (140 to 270)	0·0 (0·0 to 0·0)	135·9% (56·9 to 292·7)	12·3% (−25·3 to 87·0)	14 300 (9900 to 19 900)	2·0 (1·4 to 2·7)	135·7% (56·7 to 292·4)	12·2% (−25·4 to 86·8)
Breast cancer		940 (650to 1340)	0·1 (0·1 to 0·2)	276·6% (113·1 to 491·4)	79·3% (1·5 to 181·6)	280 (200 to 380)	0·0 (0·0 to 0·1)	155·2% (51·1 to 283·8)	21·5% (−28·0 to 82·7)	20 900 (14 800 to 28 500)	2·9 (2·0 to 3·9)	157·6% (52·1 to 287·5)	22·6% (−27·6 to 84·5)
Cervical cancer		2760 (1660to 4070)	0·4 (0·2 to 0·6)	85·2% (5·6 to 225·3)	−11·8% (−49·7 to 54·9)	1170 (740 to 1720)	0·2 (0·1 to 0·2)	52·8% (−15·1 to 161·4)	−27·2% (−59·6 to 24·5)	86 000 (54 200 to 126 000)	11·8 (7·4 to 17·3)	53·4% (−14·6 to 162·1)	−27·0% (−59·4 to 24·8)
Colon and rectum cancer		510 (350to 690)	0·1 (0·1 to 0·1)	224·2% (104·3 to 398·4)	54·3% (−2·7 to 137·3)	220 (150 to 290)	0·0 (0·0 to 0·0)	121·8% (47·3 to 237·2)	5·6% (−29·9 to 60·6)	16 000 (11 400 to 21 700)	2·2 (1·6 to 3·0)	123·2% (48·4 to 239·4)	6·3% (−29·4 to 61·6)
Lip and oral cavity cancer		370 (280to 510)	0·1 (0·0 to 0·1)	144·1% (56·0 to 296·6)	16·2% (−25·8 to 88·8)	130 (100 to 180)	0·0 (0·0 to 0·0)	98·2% (28·8 to 219·1)	−5·7% (−38·7 to 51·9)	9600 (7100 to 13 600)	1·3 (1·0 to 1·9)	98·7% (29·4 to 219·6)	−5·4% (−38·4 to 52·2)
Nasopharynx cancer		1030 (640to 1590)	0·1 (0·1 to 0·2)	74·9% (−10·3 to 215·2)	−16·7% (−57·3 to 50·1)	430 (300 to 600)	0·1 (0·0 to 0·1)	29·4% (−31·2 to 109·9)	−38·4% (−67·2 to −0·1)	33 000 (23 300 to 46 600)	4·5 (3·2 to 6·4)	28·4% (−31·9 to 109·5)	−38·9% (−67·6 to −0·2)
Pancreatic cancer		50·0 (40·0to 60·0)	0·0 (0·0 to 0·0)	177·5% (93·9 to 332·2)	32·1% (−7·7 to 105·8)	40·0 (30·0 to 50·0)	0·0 (0·0 to 0·0)	149·0% (75·1 to 279·5)	18·6% (−16·6 to 80·7)	2700 (2000 to 3500)	0·4 (0·3 to 0·5)	149·1% (75·2 to 279·6)	18·6% (−16·6 to 80·7)
Malignant skin melanoma		330 (190to 550)	0·0 (0·0 to 0·1)	219·4% (98·6 to 408·3)	52·1% (−5·4 to 142·0)	80·0 (50·0 to 120)	0·0 (0·0 to 0·0)	129·6% (56·3 to 231·7)	9·3% (−25·6 to 57·9)	5700 (3900 to 9300)	0·8 (0·5 to 1·3)	131·6% (57·1 to 235·5)	10·3% (−25·2 to 59·7)
Ovarian cancer		1480 (960to 2140)	0·2 (0·1 to 0·3)	261·3% (98·4 to 520·2)	72·0% (−5·6 to 195·3)	370 (250 to 530)	0·1 (0·0 to 0·1)	190·1% (60·3 to 404·2)	38·1% (−23·7 to 140·0)	27 700 (18 200 to 39 100)	3·8 (2·5 to 5·4)	191·6% (61·4 to 405·8)	38·8% (−23·2 to 140·8)
Testicular cancer		390 (210to 640)	0·1 (0·0 to 0·1)	234·9% (37·9 to 599·7)	59·4% (−34·3 to 233·1)	100 (60·0 to 150)	0·0 (0·0 to 0·0)	100·9% (−7·8 to 287·3)	−4·3% (−56·1 to 84·4)	7300 (4300 to 11 500)	1·0 (0·6 to 1·6)	102·8% (−7·3 to 291·1)	−3·4% (−55·9 to 86·2)
Kidney cancer		5250 (3410to 7430)	0·7 (0·5 to 1·0)	159·3% (53·4 to 282·6)	23·5% (−27·0 to 82·2)	1320 (880 to 1820)	0·2 (0·1 to 0·2)	101·7% (30·2 to 193·9)	−3·9% (−38·0 to 39·9)	116 000 (76 700 to 160 000)	15·8 (10·5 to 21·8)	102·2% (30·5 to 195·0)	−3·7% (−37·9 to 40·5)
Bladder cancer		170 (110to 230)	0·0 (0·0 to 0·0)	56·0% (−6·2 to 183·2)	−25·7% (−55·3 to 34·8)	20·0 (20·0 to 30·0)	0·0 (0·0 to 0·0)	35·9% (−13·3 to 119·6)	−35·3% (−58·7 to 4·6)	1900 (1300 to 2500)	0·3 (0·2 to 0·3)	36·8% (−13·7 to 122·4)	−34·9% (−58·9 to 5·9)
Brain and CNS cancer		9320 (6680to 13 000)	1·3 (0·9 to 1·8)	81·1% (27·1 to 149·9)	−13·8% (−39·5 to 19·0)	5400 (4000 to 7100)	0·7 (0·6 to 1·0)	69·7% (18·6 to 123·7)	−19·2% (−43·5 to 6·5)	448 000 (331 000 to 587 000)	61·2 (45·3 to 80·4)	68·6% (17·6 to 122·1)	−19·7% (−44·0 to 5·7)
Thyroid cancer		2610 (1680to 3750)	0·4 (0·2 to 0·5)	197·9% (79·1 to 462·9)	41·8% (−14·7 to 168·0)	410 (300 to 550)	0·1 (0·0 to 0·1)	89·8% (22·5 to 192·7)	−9·6% (−41·7 to 39·4)	31 600 (23 300 to 43 200)	4·3 (3·2 to 5·9)	90·8% (23·4 to 191·9)	−9·2% (−41·3 to 39·0)
Hodgkin lymphoma		3620 (2220to 5140)	0·5 (0·3 to 0·7)	68·2% (−1·5 to 171·7)	−19·9% (−53·1 to 29·4)	1830 (1090 to 2660)	0·2 (0·1 to 0·4)	29·2% (−22·9 to 111·5)	−38·5% (−63·3 to 0·7)	144 000 (86 100 to 211 000)	19·8 (11·8 to 28·9)	28·4% (−23·3 to 112·0)	−38·9% (−63·5 to 0·9)
Non-Hodgkin lymphoma		17 000 (11 200to 25 400)	2·3 (1·5 to 3·5)	128·0% (29·1 to 292·9)	8·6% (−38·5 to 87·0)	8100 (5800 to 11 100)	1·1 (0·8 to 1·5)	78·5% (12·5 to 177·2)	−15·0% (−46·4 to 32·0)	661 000 (450 000 to 906 000)	90·5 (64·3 to 124·0)	77·1% (11·4 to 174·1)	−15·7% (−47·0 to 30·5)
	Burkitt lymphoma	4270 (2270to 8030)	0·6 (0·3 to 1·1)	55·4% (−25·8 to 245·9)	−26·0% (−64·7 to 64·7)	2150 (1230 to 3880)	0·3 (0·2 to 0·5)	27·2% (−33·6 to 158·5)	−39·4% (−68·4 to 23·1)	175 000 (100 000 to 318 000)	24·0 (13·7 to 43·6)	25·8% (−34·2 to 157·2)	−40·1% (−68·7 to 22·4)
	Other non-Hodgkin lymphoma	12 700 (7960to 19 200)	1·7 (1·1 to 2·6)	170·3% (50·8 to 374·0)	28·7% (−28·2 to 125·7)	5950 (4030 to 8550)	0·8 (0·6 to 1·2)	108·8% (24·6 to 262·4)	−0·6% (−40·7 to 72·5)	486 000 (328 000 to 702 000)	66·5 (44·9 to 96·0)	107·6% (23·2 to 262·7)	−1·1% (−41·4 to 72·7)
Leukaemia		22 000 (15 700to 29 200)	3·0 (2·1 to 4·0)	56·5% (5·1 to 142·1)	−25·5% (−50·0 to 15·3)	13 900 (10 400 to 18 700)	1·9 (1·4 to 2·5)	41·2% (−6·1 to 114·1)	−32·8% (−55·3 to 1·9)	1 140 000 (850 000 to 1 530 000)	156·0 (116·6 to 209·0)	40·1% (−6·8 to 113·0)	−33·3% (−55·6 to 1·4)
	Acute lymphoid leukaemia	17 400 (12 200to 24 000)	2·4 (1·7 to 3·3)	58·1% (5·8 to 148·2)	−24·7% (−49·6 to 18·2)	11 000 (7860 to 15 400)	1·5 (1·1 to 2·1)	42·1% (−7·8 to 117·7)	−32·4% (−56·1 to 3·7)	906 000 (649 000 to 1 270 000)	124·1 (88·8 to 173·9)	41·1% (−8·8 to 117·0)	−32·8% (−56·6 to 3·3)
	Acute myeloid leukaemia	2880 (1440to 5000)	0·4 (0·2 to 0·7)	45·0% (−17·7 to 166·6)	−31·0% (−60·8 to 26·9)	2130 (1080 to 3610)	0·3 (0·1 to 0·5)	41·3% (−17·4 to 168·0)	−32·7% (−60·7 to 27·6)	172 000 (87 100 to 293 000)	23·5 (11·9 to 40·2)	39·5% (−19·0 to 164·9)	−33·6% (−61·5 to 26·1)
	Chronic myeloid leukaemia	250 (100to 490)	0·0 (0·0 to 0·1)	2·1% (−63·7 to 179·2)	−51·4% (−82·7 to 32·9)	180 (70·0 to 330)	0·0 (0·0 to 0·1)	−2·8% (−64·2 to 164·5)	−53·7% (−82·9 to 26·0)	14 500 (6000 to 26 800)	2·0 (0·8 to 3·7)	−3·6% (−64·7 to 162·2)	−54·1% (−83·2 to 24·8)
	Other leukaemia	1420 (660to 2470)	0·2 (0·1 to 0·3)	80·4% (−18·9 to 298·0)	−14·1% (−61·4 to 89·5)	570 (260 to 970)	0·1 (0·0 to 0·1)	42·6% (−27·4 to 226·0)	−32·1% (−65·4 to 55·2)	47 000 (21 500 to 79 800)	6·4 (2·9 to 10·9)	41·8% (−28·5 to 227·4)	−32·5% (−66·0 to 55·9)
Eye cancer		3080 (1350to 6320)	0·4 (0·2 to 0·9)	23·5% (−66·5 to 335·1)	−41·2% (−84·1 to 107·1)	2390 (860 to 5240)	0·3 (0·1 to 0·7)	4·7% (−74·5 to 383·0)	−50·2% (−87·9 to 130·0)	208 000 (74 800 to 456 000)	28·5 (10·2 to 62·4)	4·8% (−74·7 to 386·2)	−50·1% (−87·9 to 131·5)
	Retinoblastoma	2630 (940to 5890)	0·4 (0·1 to 0·8)	17·0% (−73·5 to 504·2)	−44·3% (−87·4 to 187·7)	2300 (810 to 5130)	0·3 (0·1 to 0·7)	3·5% (−76·2 to 425·8)	−50·7% (−88·7 to 150·3)	200 000 (70 900 to 448 000)	27·4 (9·7 to 61·3)	3·7% (−76·2 to 426·1)	−50·6% (−88·7 to 150·5)
	Other eye cancers	450 (250to 840)	0·1 (0·0 to 0·1)	84·7% (−16·8 to 295·6)	−12·1% (−60·4 to 88·3)	100 (50·0 to 160)	0·0 (0·0 to 0·0)	41·1% (−32·6 to 159·0)	−32·8% (−67·9 to 23·3)	7500 (3900 to 12 400)	1·0 (0·5 to 1·7)	41·8% (−31·2 to 154·8)	−32·5% (−67·2 to 21·3)
Soft tissue and other extraosseous sarcomas		7430 (4310to 11 700)	1·0 (0·6 to 1·6)	76·4% (−13·4 to 191·9)	−16·0% (−58·8 to 39·0)	3410 (2000 to 5070)	0·5 (0·3 to 0·7)	53·5% (−17·7 to 154·5)	−26·9% (−60·8 to 21·2)	282 000 (165 000 to 419 000)	38·6 (22·6 to 57·3)	52·2% (−19·1 to 152·7)	−27·5% (−61·5 to 20·3)
Malignant neoplasm of bone and articular cartilage		9210 (4580to 17 900)	1·3 (0·6 to 2·5)	99·9% (−15·9 to 366·3)	−4·8% (−60·0 to 122·0)	4900 (2600 to 7800)	0·7 (0·4 to 1·1)	69·8% (−14·2 to 191·1)	−19·2% (−59·2 to 38·6)	380 000 (203 000 to 607 000)	52·0 (27·7 to 83·1)	68·9% (−15·1 to 192·9)	−19·6% (−59·6 to 39·5)
Neuroblastoma and other peripheral nervous cell tumours		1980 (1120to 3330)	0·3 (0·1 to 0·5)	167·1% (40·8 to 399·3)	27·2% (−33·0 to 137·7)	790 (480 to 1230)	0·1 (0·1 to 0·2)	108·5% (23·7 to 234·6)	−0·7% (−41·1 to 59·3)	67 600 (41 100 to 106 000)	9·3 (5·6 to 14·6)	107·9% (22·7 to 235·1)	−1·0% (−41·6 to 59·6)
Other malignant neoplasms		9980 (7270to 12 900)	1·4 (1·0 to 1·8)	40·7% (−14·2 to 118·0)	−33·0% (−59·2 to 3·8)	5900 (4200 to 7500)	0·8 (0·6 to 1·0)	31·3% (−19·4 to 101·0)	−37·5% (−61·6 to −4·3)	489 000 (353 000 to 632 000)	67·0 (48·3 to 86·5)	30·2% (−20·1 to 100·4)	−38·0% (−62·0 to −4·6)

Data in parentheses are 95% uncertainty intervals. DALY=disability-adjusted life-year.

The top five childhood cancer incidences in Africa in 2023 were leukaemia, non-Hodgkin lymphoma, other malignant neoplasms, brain and CNS cancers, and malignant neoplasms of bone and articular cartilage. Detailed sex-specific burden estimates are provided in the appendix (pp 128–133).

In 2023, cancers in adolescents and young adults (ages 15–39 years) in Africa accounted for 372 000 (95%UI 309 000–45 000) incident cases, equivalent to 24·2% (22·6–25·8) of all cancer cases; those in adults aged 40–64 years accounted for 681 000 (599 000–780 000) cases, equivalent to 44·2% (43·7–44·6); and those in adults aged 65 years and older accounted for 401 000 (346 000–456 000) cases, equivalent to 26·0% (25·3–26·1) of all cancer cases (figure 2; GBD results tool).

**Figure 2:**
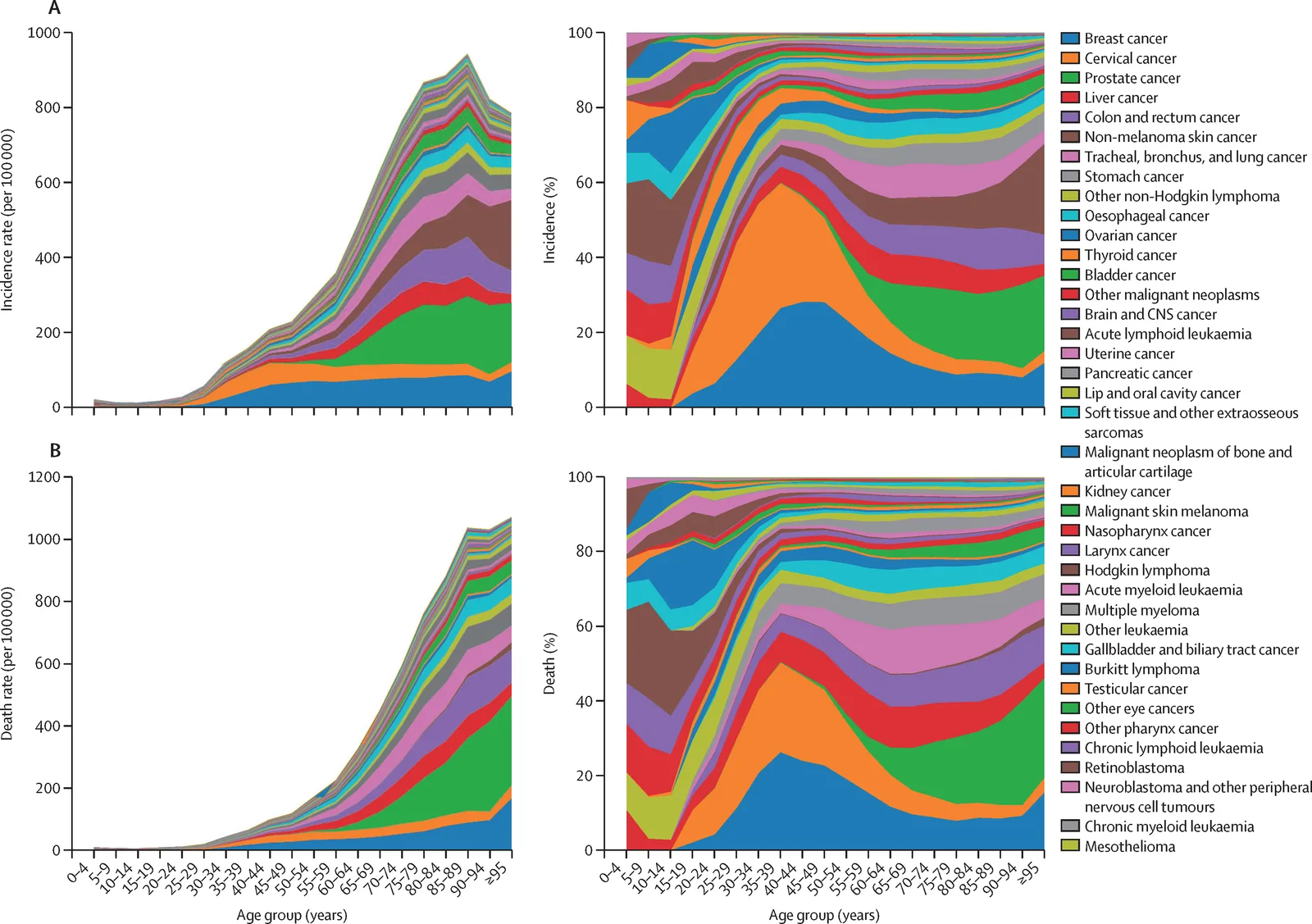
Age-specific cancer incidence (A) and deaths (B) for all sexes combined in Africa, 2023 Results are presented by 5-year age group. Left panels present age-specific incidence and death rates. Right panels present age-specific new case and death proportions. Several causes are not presented to not duplicate burden: eye cancer (level 3); non-Hodgkin lymphoma (level 3); leukaemia (level 3); and liver cancer due to hepatitis B, liver cancer due to hepatitis C, liver cancer due to alcohol use, liver cancer due to non-alcoholic steatohepatitis, liver cancer due to other causes, and hepatoblastoma (level 4).

Cancer deaths in Africa increased from 324 000 (95% UI 288 000–359 000) in 1990 to 526 000 (477 000–575 000) in 2010, 748 000 (677 000–820 000) in 2020 (see GBD results tool for 2020 data), and 923 000 (824 000–1 020 000) in 2023 (920 000 [822 000–1 010 000] when excluding non-melanoma skin cancer; GBD results tool) across all ages and both sexes. The age-standardised death rate rose from 98·9 (88·5–110·4) per 100 000 in 1990 to 98·4 (88·6–108·4) per 100 000 in 2010, 102·2 (91·3–111·5) per 100 000 in 2020, and 114·2 (100·6–125·8) per 100 000 in 2023. The age-standardised death rate from cancer in Africa did not peak during the COVID-19 pandemic. Rates were 102·2 (91·3–111·5) per 100 000 in 2020, 106·5 (95·4–116·8) per 100 000 in 2021, and 110·6 (98·8–121·3) per 100 000 in 2022, across all ages and both sexes (GBD results tool). These changes correspond to relative increases of 15·5% (2·3–31·9) between 1990 and 2023 and 11·7% (6·6–16·4) between 2020 and 2023, the latter coinciding with the COVID-19 pandemic (Table 1, Table 2, Table 3; appendix p 134; GBD results tool).

In 2023, the highest age-standardised death rates were observed in Equatorial Guinea (178·6 [95% UI 143·9–208·1] per 100 000) and Ethiopia (169·9 [147·5–190·5] per 100 000), whereas the lowest were in Algeria (62·6 [47·9–77·6] per 100 000), The Gambia (58·8 [47·7–71·2] per 100 000), and Comoros (53·0 [45·9–60·2] per 100 000). Between 2010 and 2023, most countries showed increases, with the largest relative rises in Côte d’Ivoire (49·2% [27·6–78·6]), Ghana (38·3% [18·0–63·2]), Gabon (37·4% [18·4–59·8]), and Equatorial Guinea (37·0% [11·5–64·9]). Six countries had a change in age-standardised death rates—Tunisia (–5·2% [–16·3 to 7·9]), Libya (–5·3% [–18·5 to 8·9]), Zimbabwe (–5·4% [–18·1 to 10·2]), Seychelles (–6·5% [–14·2 to 3·8]), Tanzania (–9·9% [–22·7 to 6·2]), and Eswatini (–11·2% [–27·7 to 6·4])—although none were statistically significant. Country-level estimates and estimates by sex are presented in Table 1, Table 3, figure 1, and the appendix (pp 134–147).

In 1990, the five leading causes of cancer death across all ages and sexes in Africa were liver cancer, cervical cancer, prostate cancer, stomach cancer, and breast cancer. In 2023, breast cancer (129 000 [95% UI 94 000–166 000]), cervical cancer (107 000 [75 000–151 000]), liver cancer (87 000 [59 000–122 000]), tracheal, bronchus and lung cancer (72 000 [56 000–92 000]), and colorectal cancer (68 000 [52 000–86 000]) were the leading causes of cancer mortality (table 2; appendix pp 148–151). In females, cervical cancer, breast cancer, liver cancer, and stomach cancer were the top causes in 1990. By 2010, cervical cancer, breast cancer, liver cancer, and colorectal cancer predominated. In 2023, breast cancer, cervical cancer, colorectal cancer, and liver cancer were the leading causes of cancer mortality among females in Africa; together, breast and cervical cancer accounted for 45·9% (37·3–54·7) of all female cancer deaths (appendix pp 152–155). In males, liver cancer; prostate cancer; tracheal, bronchus, and lung cancer; and stomach cancer were leading in 1990. In 2010, prostate cancer; liver cancer; tracheal, bronchus, lung cancer; and colorectal cancer were the most common causes of cancer mortality among males in Africa. This pattern persisted in 2023. Together, these four cancers accounted for 51·2% (95% UI 41·1–62·6) of all male cancer deaths (appendix pp 156–159).

Between 1990 and 2023, the largest increases in age-standardised mortality rates among women were observed for pancreatic cancer (82·1% [95% UI 18·8–182·2], breast cancer (86·9% [14·5–172·2]), ovarian cancer (94·3% [16·7–209·3]), and multiple myeloma (103·1% [20·4–263·8]) across all ages. Detailed mortality estimates by sex and year are presented in table 2, figure 1, figure 3, and the appendix (pp 148–159).

**Figure 3:**
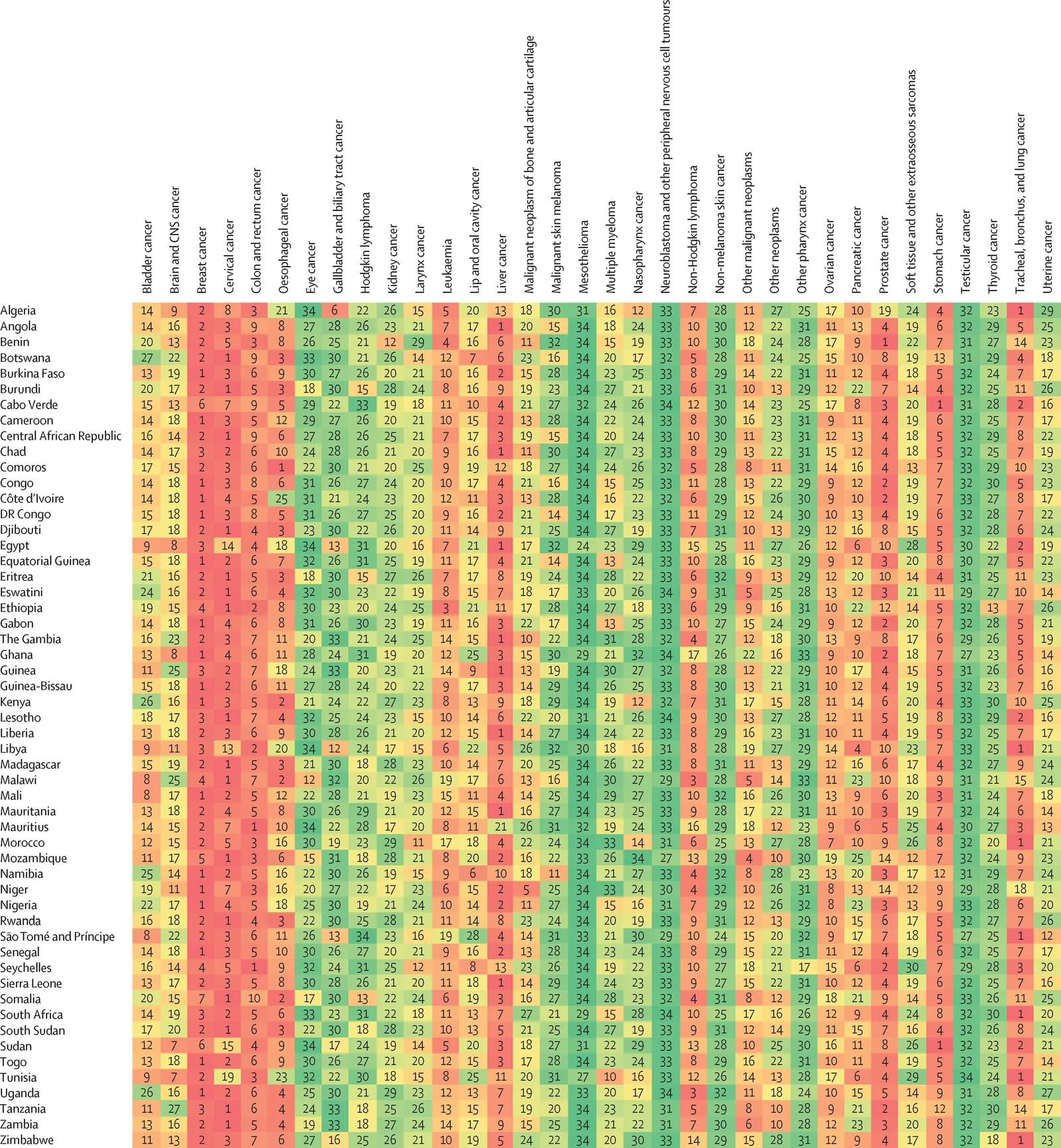
Death rank by age-standardised death rate per 100 000 for both sexes in 54 countries in Africa, 2023 Cells are coloured according to rank, ranging from highest rank (red) to lowest rank (green).

Childhood cancer deaths in Africa (ages 0–19 years) increased from 36 400 (95% UI 25 800–43 700) in 1990 to 41 600 (36 400–47 800) in 2010, and 54 700 (47 500–64 500) in 2023. Africa contributed 38·0% (36·3–40·0) of 144 000 (131 000–162 000) childhood cancer deaths globally in 2023 (GBD results tool). The age-specific death rate declined from 10·5 (8·3–12·6) per 100 000 in 1990 to 7·5 (6·5–8·8) per 100 000 in 2023 for both sexes.

The top five childhood cancer deaths in Africa in 2023 were leukaemia (13 900 [95% UI 10 400–18 700]); non-Hodgkin lymphoma (8100 [5800–11 100]); other malignant neoplasms (5900 [4200–7500]); brain and CNS cancers (5400 [4000–7100]); and malignant neoplasms of bone and articular cartilage (4900 [2600–7800]). Detailed sex-specific childhood cancer burden estimates are provided in table 3 and the appendix (pp 128–133).

In 2023, cancers in adolescents and young adults (ages 15–39 years) in Africa accounted for 150 000 (95% UI 133 000–170 000) deaths, equivalent to 16·3% (16·1–16·8) of all cancer deaths; cancers in adults aged 40–64 years accounted for 397 000 (351 000–441 000) deaths, equivalent to 43·0% (42·6–43·2); and cancers in adults aged 65 years and older accounted for 334 000 (284 000–382 000) deaths, equivalent to 36·2% (34·5–37·5) of total cancer deaths (figure 2; GBD results tool).

Cancer DALYs in Africa increased from 12·9 million (95% UI 11·4–14·5) in 1990 to 27·7 million (25·4–30·2) in 2020 and 33·9 million (30·6–37·1) in 2023 (33·9 million [30·5–37·0] when excluding non-melanoma skin cancer) across all ages and both sexes, of which 98·2% (97·9–98·3) were attributable to YLLs and 1·8% (1·7–2·1) to YLDs. Detailed DALY and YLL estimates by country and year are presented in table 1 and the appendix (pp 160–179).

In 1990, the leading causes of cancer-related DALYs in Africa across all ages and sexes were liver cancer, cervical cancer, leukaemia, breast cancer, and stomach cancer. By 2010, cervical cancer, breast cancer, liver cancer, leukaemia, and non-Hodgkin lymphoma predominated. In 2023, breast cancer (5·05 million [95% UI 3·67–6·51]), cervical cancer (4·57 million [3·19–6·47]), liver cancer (3·02 million [2·04–4·29]), leukaemia (2·10 million [1·63–2·78]), and colorectal cancer (2·04 million [1·58–2·56]) were the leading causes of DALYs across both sexes and all ages (appendix pp 180–183).

In females, cervical cancer, breast cancer, leukaemia, and liver cancer were the leading causes in 1990. In 2023, breast cancer, cervical cancer, colorectal cancer, and liver cancer were most prominent; together, breast and cervical cancer accounted for 47·6% (95% UI 37·8–57·3) of all female cancer DALYs. By 2023, liver cancer; tracheal, bronchus, and lung cancer; prostate cancer; and leukaemia were predominant in males; tracheal, bronchus, and lung cancer, prostate cancer, and colorectal cancer together accounted for 42·3% (33·0–52·2) of all male cancer DALYs (appendix pp 184–191).

Between 2010 and 2023, significant increases in age-standardised DALYs were observed for ovarian cancer (55·5 [95% UI 39·3–75·5] per 100 000 in 2010 to 93·2 [66·2–125·4] in 2023 per 100 000), breast cancer (318·4 [246·1–423·8] per 100 000 to 509·2 [372·0–657·0] per 100 000), multiple myeloma (21·7 [15·9–29·0] per 100 000 to 31·1 [23·0–42·0] per 100 000), and malignant skin melanoma (16·7 [10·9–23·8] per 100 000 to 23·6 [15·6–33·7] per 100 000). Detailed DALY estimates by sex and year are presented in table 2 and the appendix (pp 172–182).

Childhood cancer DALYs in Africa (ages 0–19 years) increased from 3·0 million (95% UI 2·4–3·6) in 1990 to 3·4 million (3·0–3·9) in 2010, and 4·5 million (3·9–5·3) in 2023. In 2023, Africa contributed 38·0% (36·4–40·2) of 11·7 million (10·7–13·2) DALYs due to childhood cancer globally in both sexes. The age-specific DALY rate declined from 863·6 (680·3–1041·1) per 100 000 in 1990 to 612·4 (533·3–723·4) per 100 000 in 2023 (table 3; appendix pp 125–131).

Cancers in adolescents and young adults (ages 15–39 years) contributed 9·02 million (95% UI 8·08–10·2) DALYs, equivalent to 26·6% (26·4–27·5) of total cancer DALYs in Africa; those in adults aged 40–64 years contributed 15·2 million (13·4–16·9) DALYs, equivalent to 44·8% (43·8–45·6); and those in adults aged 65 years and older contributed 6·22 million (5·32–7·09) DALYs, equivalent to 18·3% (17·4–19·1; GBD results tool).

In 2023, 10·7 million (95% UI 8·9–12·9) DALYs and 306 000 (254 000–363 000) cancer deaths in Africa were attributable to the 88 risk factors assessed in GBD 2023, equivalent to 31·6% (26·8–35·9) of the total 33·9 million (30·6–37·1) DALYs and 33·1% (28·7–37·2) of the total 923 000 (824 000–1 020 000) cancer deaths (table 4). Between 1990 and 2023, deaths attributable to the risk factors increased by 216·0% (159·3–284·5). Over the same period, the age-standardised death rate attributable to the risk factor rose by 22·0% (2·0–48·6) across all ages and both sexes.

**Table 4:** Death and DALY counts and age-standardised rates of cancer attributable to risk factors in 2023 and percentage change in counts and age-standardised rates between 1990 and 2023 in Africa for all ages and sexes combined

			**Deaths**						**DALYs**					
			Counts, hundreds, 1990	Age-standardised rate, 1990	Counts, hundreds, 2023	Age-standardised rate, 2023	Percentage change in count, 1990–2023	Percentage change in age-standardised rate, 1990–2023	Counts, hundreds, 1990	Age-standardisedrate, 1990	Counts, hundreds, 2023	Age-standardised rate, 2023	Percentage change in count, 1990–2023	Percentage change in age-standardised rate, 1990–2023
All risk factors			968 (784 to 1170)	30·9 (25·0 to 36·5)	3060 (2540 to 3630)	37·7 (31·4 to 44·4)	216·0% (159·3 to 284·5)	22·0% (2·0 to 48·6)	3420 (2680 to 4230)	952·7 (767·1 to 1156·7)	10 700 (8900 to 12 900)	1155·0 (957·3 to 1369·4)	213·0% (147·7 to 284·2)	21·2% (−1·1 to 47·4)
Environmental or occupational risks			90·4 (61·7 to 128)	3·2 (2·2 to 4·5)	266 (182 to 353)	3·7 (2·5 to 4·9)	194·7% (129·3 to 313·5)	15·8% (−10·6 to 65·0)	265 (183 to 371)	83·5 (57·2 to 117·4)	766 (529 to 1010)	94·5 (64·4 to 124·9)	189·3% (126·8 to 304·6)	13·2% (−11·8 to 58·3)
	Air pollution		65·3 (39·9 to 95·8)	2·3 (1·4 to 3·4)	184 (117 to 262)	2·6 (1·6 to 3·7)	182·4% (115·8 to 311·0)	11·1% (−15·8 to 62·9)	189 (116 to 276)	60·2 (36·9 to 88·1)	524 (331 to 740)	65·3 (41·3 to 92·3)	177·6% (112·8 to 300·5)	8·4% (−16·7 to 57·0)
		Particulate matter pollution	65·3 (39·9 to 95·8)	2·3 (1·4 to 3·4)	184 (117 to 262)	2·6 (1·6 to 3·7)	182·4% (115·8 to 311·0)	11·1% (−15·8 to 62·9)	189 (116 to 276)	60·2 (36·9 to 88·1)	524 (331 to 740)	65·3 (41·3 to 92·3)	177·6% (112·8 to 300·5)	8·4% (−16·7 to 57·0)
	Ambient particulate matter pollution		20·4 (11·8 to 30·8)	0·7 (0·4 to 1·1)	93·1 (56·4 to 133)	1·3 (0·8 to 1·9)	356·3% (241·6 to 572·6)	80·4% (34·9 to 165·9)	58·3 (33·8 to 88·0)	18·8 (10·9 to 28·3)	257 (156 to 367)	32·7 (19·8 to 46·7)	341·5% (231·0 to 538·8)	74·3% (30·5 to 155·8)
	Household air pollution from solid fuels		45·0 (27·2 to 68·8)	1·6 (1·0 to 2·4)	91·7 (58·1 to 130)	1·3 (0·8 to 1·8)	104·0% (48·7 to 207·4)	−20·5% (−41·4 to 20·5)	131 (79·0 to 199)	41·5 (25·0 to 63·4)	268 (172 to 377)	32·7 (20·8 to 46·3)	104·9% (49·9 to 206·0)	−21·2% (−42·4 to 18·2)
	Other environmental risks		8·90 (−3·80 to 23·1)	0·3 (−0·1 to 0·8)	27·5 (−11·2 to 74·4)	0·4 (−0·2 to 1·0)	207·5% (134·1 to 345·2)	20·9% (−7·3 to 76·2)	25·6 (−10·9 to 66·4)	8·2 (−3·5 to 21·1)	77·3 (−32·1 to 213)	9·7 (−4·0 to 26·3)	201·8% (125·9 to 336·0)	18·4% (−10·4 to 71·0)
		Residential radon	8·90 (−3·80 to 23·1)	0·3 (−0·1 to 0·8)	27·5 (−11·2 to 74·4)	0·4 (−0·2 to 1·0)	207·5% (134·1 to 345·2)	20·9% (−7·3 to 76·2)	25·6 (−10·9 to 66·4)	8·2 (−3·5 to 21·1)	77·3 (−32·1 to 213)	9·7 (−4·0 to 26·3)	201·8% (125·9 to 336·0)	18·4% (−10·4 to 71·0)
Occupational risks			24·9 (16·3 to 34·7)	0·9 (0·6 to 1·2)	77·6 (52·5 to 107)	1·0 (0·7 to 1·4)	211·4% (148·4 to 324·7)	21·7% (−3·0 to 67·6)	75·6 (51·0 to 104)	23·3 (15·6 to 32·3)	231 (159 to 319)	27·9 (19·0 to 38·6)	206·1% (146·4 to 309·1)	19·9% (−4·2 to 63·1)
	Occupational carcinogens		24·9 (16·3 to 34·7)	0·9 (0·6 to 1·2)	77·6 (52·5 to 107)	1·0 (0·7 to 1·4)	211·4% (148·4 to 324·7)	21·7% (−3·0 to 67·6)	75·6 (51·0 to 104)	23·3 (15·6 to 32·3)	231 (159 to 319)	27·9 (19·0 to 38·6)	206·1% (146·4 to 309·1)	19·9% (−4·2 to 63·1)
	Occupational exposure to asbestos		12·4 (6·90 to 20·3)	0·5 (0·3 to 0·8)	36·3 (22·1 to 57·3)	0·6 (0·3 to 0·9)	193·8% (127·7 to 299·5)	15·2% (−10·7 to 57·8)	29·3 (16·3 to 48·1)	10·6 (5·9 to 17·3)	84·8 (51·5 to 132)	12·1 (7·3 to 19·1)	189·0% (121·5 to 294·0)	14·3% (−11·7 to 54·4)
	Occupational exposure to arsenic		0·800 (0·300 to 1·40)	0·0 (0·0 to 0·0)	2·50 (1·00 to 4·30)	0·0 (0·0 to 0·1)	208·9% (136·0 to 350·2)	21·4% (−7·4 to 78·0)	2·70 (1·00 to 4·70)	0·8 (0·3 to 1·4)	8·20 (3·30 to 14·1)	0·9 (0·4 to 1·6)	201·9% (129·5 to 336·1)	16·8% (−10·9 to 69·6)
	Occupational exposure to benzene		1·00 (0·300 to 1·70)	0·0 (0·0 to 0·0)	2·90 (0·90 to 4·90)	0·0 (0·0 to 0·0)	185·5% (102·2 to 292·6)	13·6% (−19·2 to 56·7)	5·60 (1·60 to 9·50)	1·1 (0·3 to 1·8)	15·8 (4·90 to 27·1)	1·2 (0·4 to 2·0)	180·4% (98·9 to 286·6)	12·0% (−20·5 to 54·0)
	Occupational exposure to beryllium		0 (0 to 0·100)	0·0 (0·0 to 0·0)	0·100 (0·100 to 0·200)	0·0 (0·0 to 0·0)	221·3% (142·9 to 361·9)	25·8% (−4·8 to 80·2)	0·100 (0·100 to 0·200)	0·0 (0·0 to 0·1)	0·400 (0·300 to 0·600)	0·0 (0·0 to 0·1)	215·6% (137·5 to 355·9)	21·7% (−8·1 to 75·5)
	Occupational exposure to cadmium		0·100 (0·100 to 0·100)	0·0 (0·0 to 0·0)	0·200 (0·200 to 0·300)	0·0 (0·0 to 0·0)	213·6% (138·6 to 356·5)	23·1% (−6·3 to 78·3)	0·300 (0·200 to 0·400)	0·1 (0·1 to 0·1)	0·800 (0·600 to 1·10)	0·1 (0·1 to 0·1)	207·0% (131·9 to 347·9)	18·7% (−10·1 to 72·5)
	Occupational exposure to chromium		0·200 (0·100 to 0·200)	0·0 (0·0 to 0·0)	0·500 (0·400 to 0·700)	0·0 (0·0 to 0·0)	233·7% (153·1 to 379·6)	31·1% (−0·5 to 89·6)	0·600 (0·400 to 0·700)	0·2 (0·1 to 0·2)	1·80 (1·30 to 2·30)	0·2 (0·2 to 0·3)	226·3% (146·1 to 366·9)	26·2% (−4·6 to 80·5)
	Occupational exposure to diesel engine exhaust		2·40 (1·80 to 3·20)	0·1 (0·1 to 0·1)	9·10 (6·70 to 12·3)	0·1 (0·1 to 0·1)	277·4% (184·4 to 445·8)	48·3% (11·7 to 116·3)	8·20 (6·10 to 10·9)	2·4 (1·8 to 3·2)	30·1 (22·3 to 40·2)	3·5 (2·5 to 4·7)	269·2% (175·9 to 431·7)	42·9% (7·5 to 106·4)
	Occupational exposure to formaldehyde		0·600 (0·500 to 0·800)	0·0 (0·0 to 0·0)	1·70 (1·30 to 2·20)	0·0 (0·0 to 0·0)	172·7% (100·2 to 261·7)	7·4% (−21·3 to 43·7)	3·40 (2·50 to 4·40)	0·7 (0·5 to 0·9)	9·00 (6·70 to 11·8)	0·7 (0·5 to 0·9)	166·2% (96·1 to 253·9)	5·6% (−22·3 to 40·6)
	Occupational exposure to nickel		0·900 (0·200 to 2·10)	0·0 (0·0 to 0·1)	2·80 (0·600 to 6·80)	0·0 (0·0 to 0·1)	199·8% (128·2 to 340·3)	17·9% (−10·2 to 73·6)	3·20 (0·70 to 7·20)	0·9 (0·2 to 2·1)	9·30 (2·00 to 22·5)	1·1 (0·2 to 2·6)	192·7% (122·4 to 328·2)	13·4% (−13·8 to 66·1)
	Occupational exposure to polycyclic aromatic hydrocarbons		0·600 (0·400 to 0·800)	0·0 (0·0 to 0·0)	2·00 (1·50 to 2·70)	0·0 (0·0 to 0·0)	238·8% (157·5 to 391·0)	33·1% (0·9 to 92·7)	2·00 (1·40 to 2·70)	0·6 (0·4 to 0·8)	6·70 (5·10 to 9·10)	0·8 (0·6 to 1·0)	231·1% (152·2 to 376·0)	28·1% (−2·6 to 85·1)
	Occupational exposure to silica		5·50 (2·40 to 9·10)	0·2 (0·1 to 0·3)	18·3 (8·10 to 30·4)	0·2 (0·1 to 0·4)	230·8% (146·6 to 392·5)	30·5% (−2·9 to 94·7)	18·7 (7·90 to 30·7)	5·5 (2·3 to 9·1)	60·1 (26·4 to 99·6)	6·9 (3·0 to 11·5)	221·7% (138·5 to 378·7)	25·1% (−6·8 to 86·0)
	Occupational exposure to sulfuric acid		1·00 (0·400 to 1·90)	0·0 (0·0 to 0·1)	2·60 (1·00 to 5·20)	0·0 (0·0 to 0·1)	171·9% (71·8 to 287·0)	5·1% (−33·2 to 50·1)	3·40 (1·40 to 6·80)	1·0 (0·4 to 2·0)	9·20 (3·60 to 18·3)	1·0 (0·4 to 2·0)	170·5% (69·6 to 281·5)	3·1% (−35·2 to 46·2)
	Occupational exposure to trichloroethylene		0 (0 to 0)	0·0 (0·0 to 0·0)	0 (0 to 0·100)	0·0 (0·0 to 0·0)	259·4% (154·4 to 417·8)	38·6% (−1·5 to 100·5)	0 (0 to 0·100)	0·0 (0·0 to 0·0)	0·100 (0 to 0·300)	0·0 (0·0 to 0·0)	259·8% (156·4 to 412·9)	36·6% (−3·0 to 96·0)
Behavioural risks			844 (647 to 1040)	26·6 (20·6 to 32·3)	2490 (2000 to 3060)	29·9 (24·4 to 36·5)	195·0% (130·9 to 266·7)	12·7% (−10·0 to 41·3)	3050 (2290 to 3870)	836·5 (640·0 to 1041·7)	9020 (7180 to 11 200)	949·6 (761·3 to 1172·5)	195·9% (124·8 to 282·2)	13·5% (−12·0 to 40·6)
	Tobacco use		273 (197 to 360)	9·5 (6·9 to 12·6)	720 (550 to 940)	9·6 (7·5 to 12·8)	161·4% (99·9 to 251·1)	1·0% (−24·1 to 39·0)	811 (581 to 1060)	256·5 (184·8 to 337·5)	2120 (1660 to 2740)	258·7 (200·5 to 338·3)	161·3% (100·3 to 244·2)	0·8% (−22·4 to 34·1)
		Smoking	244 (172 to 330)	8·6 (6·1 to 11·6)	609 (450 to 841)	8·3 (6·2 to 11·5)	148·9% (82·5 to 243·7)	−3·1% (−29·8 to 36·9)	708 (495 to 954)	227·5 (159·8 to 305·9)	1740 (1300 to 2390)	218·0 (161·7 to 300·2)	145·5% (81·0 to 235·0)	−4·2% (−29·5 to 31·4)
		Chewing tobacco	7·30 (2·90 to 14·6)	0·2 (0·1 to 0·5)	17·5 (7·4 to 34·0)	0·2 (0·1 to 0·4)	139·1% (−8·0 to 491·0)	−6·9% (−64·2 to 139·4)	24·7 (9·80 to 48·8)	7·2 (2·8 to 14·3)	57·0 (24·9 to 108)	6·5 (2·8 to 12·5)	131·0% (−10·2 to 440·2)	−9·9% (−65·3 to 117·5)
		Second–hand smoke	33·4 (23·8 to 44·5)	1·1 (0·8 to 1·5)	118 (89·3 to 150)	1·5 (1·1 to 1·9)	253·6% (170·8 to 373·0)	33·1% (1·0 to 79·2)	112 (79·6 to 150)	32·7 (23·4 to 43·7)	406 (298 to 535)	44·7 (33·8 to 57·7)	263·8% (179·4 to 386·9)	36·5% (4·9 to 82·6)
	High alcohol use		111 (69·5 to 166)	3·6 (2·3 to 5·5)	272 (176 to 395)	3·4 (2·2 to 4·8)	144·7% (81·3 to 249·6)	−6·3% (−32·1 to 33·6)	362 (226 to 545)	107·7 (66·6 to 161·5)	903 (583 to 1310)	101·7 (65·7 to 146·9)	149·5% (86·4 to 253·6)	−5·6% (−29·7 to 33·3)
	Drug use		2·70 (1·30 to 4·80)	0·1 (0·0 to 0·1)	19·7 (11·5 to 33·7)	0·3 (0·1 to 0·4)	626·4% (357·8 to 1095·5)	192·1% (83·2 to 379·1)	9·30 (4·50 to 16·2)	2·7 (1·3 to 4·8)	64·2 (36·2 to 114)	7·3 (4·2 to 12·6)	590·9% (332·8 to 1033·4)	171·0% (70·2 to 345·8)
	Dietary risks		153 (58·1 to 257)	5·2 (2·2 to 8·5)	500 (150 to 850)	6·4 (2·2 to 10·4)	225·0% (116·6 to 320·9)	22·4% (−18·7 to 62·0)	503 (162 to 867)	148·5 (52·6 to 251·9)	1670 (435 to 2920)	185·4 (52·3 to 320·0)	231·8% (119·1 to 323·8)	24·9% (−16·6 to 60·9)
	Diet low in fruits		17·3 (8·1 to 28·8)	0·6 (0·3 to 1·0)	47·5 (22·4 to 80·2)	0·7 (0·3 to 1·1)	174·8% (64·8 to 397·7)	7·3% (−35·3 to 95·4)	50·4 (23·6 to 84·0)	16·0 (7·5 to 26·7)	137 (64·0 to 227)	16·8 (7·9 to 28·3)	171·2% (63·4 to 388·2)	5·2% (−36·7 to 89·9)
	Diet low in vegetables		41·1 (−7·50 to 78·9)	1·4 (−0·3 to 2·8)	105 (−18·7 to 206)	1·4 (−0·3 to 2·8)	155·1% (85·4 to 251·5)	−1·4% (−28·0 to 34·9)	124 (−22·3 to 238)	38·8 (−7·0 to 74·4)	313 (−55·6 to 612)	37·7 (−6·7 to 73·9)	152·4% (81·3 to 256·0)	−2·9% (−29·8 to 35·2)
	Diet low in wholegrains		16·7 (6·30 to 28·0)	0·6 (0·2 to 1·0)	59·1 (21·2 to 102)	0·8 (0·3 to 1·4)	253·6% (85·2 to 582·8)	36·6% (−30·5 to 167·5)	52·8 (20·0 to 88·1)	15·8 (6·0 to 26·4)	184 (67·3 to 315)	21·4 (7·7 to 36·9)	247·9% (91·9 to 563·0)	35·0% (−28·3 to 159·9)
	Diet low in milk		20·2 (9·00 to 30·6)	0·7 (0·1 to 1·0)	79·1 (41·5 to 114)	1·0 (0·4 to 1·5)	291·1% (86·4 to 854·9)	49·4% (−66·8 to 439·6)	71·8 (45·2 to 110)	20·2 (11·0 to 30·4)	273 (147 to 418)	29·7 (16·7 to 43·6)	280·5% (132·9 to 581·7)	46·9% (−15·3 to 220·0)
	Diet high in processed meat		3·30 (0·900 to 6·00)	0·1 (0·0 to 0·2)	13·0 (4·30 to 22·3)	0·2 (0·1 to 0·3)	298·6% (145·1 to 509·4)	53·6% (−4·4 to 136·2)	10·4 (2·90 to 19·4)	3·1 (0·9 to 5·8)	41·2 (13·5 to 71·1)	4·7 (1·6 to 8·2)	294·1% (138·5 to 520·5)	51·9% (−7·0 to 133·9)
	Diet low in fibre		0·600 (0·200 to 1·60)	0·0 (0·0 to 0·1)	1·40 (0·300 to 3·30)	0·0 (0·0 to 0·0)	121·1% (−31·4 to 585·2)	−18·1% (−74·6 to 151·8)	1·90 (0·600 to 4·90)	0·6 (0·2 to 1·5)	4·30 (0·900 to 10·3)	0·5 (0·1 to 1·2)	125·3% (−28·4 to 613·7)	−15·5% (−73·7 to 165·0)
	Diet high in sodium		15·4 (0 to 91·5)	0·5 (0·0 to 3·2)	32·8 (0 to 201)	0·4 (0·0 to 2·8)	112·2% (−80·7 to 605·4)	−17·6% (−91·9 to 190·5)	48·4 (0 to 289)	14·5 (0·0 to 86·0)	101 (0 to 607)	11·7 (0·0 to 71·4)	107·8% (−84·1 to 520·9)	−19·3% (−93·1 to 157·2)
	Diet low in calcium		36·5 (24·7 to 49·6)	1·3 (0·9 to 1·8)	101 (65·7 to 137)	1·4 (0·9 to 1·9)	176·1% (84·3 to 324·8)	5·2% (−29·6 to 63·5)	114 (79·1 to 153)	34·6 (23·4 to 46·6)	314 (216 to 424)	36·2 (23·7 to 48·8)	174·4% (88·1 to 309·3)	4·8% (−29·8 to 60·4)
	Low physical activity		13·0 (6·10 to 19·1)	0·5 (0·2 to 0·7)	55·1 (25·0 to 84·6)	0·8 (0·4 to 1·2)	324·4% (224·3 to 434·8)	55·7% (19·7 to 97·2)	36·6 (16·0 to 55·7)	12·0 (5·5 to 17·8)	161 (65·3 to 253)	19·6 (8·5 to 30·5)	341·3% (235·0 to 466·2)	64·2% (24·8 to 108·4)
	Unsafe sex		352 (227 to 530)	9·6 (6·3 to 14·6)	1080 (750 to 1510)	11·3 (7·8 to 15·7)	205·4% (93·2 to 408·2)	17·2% (−25·1 to 95·0)	1520 (974 to 2310)	368·6 (238·1 to 555·6)	4570 (3190 to 6470)	430·7 (302·9 to 602·7)	200·5% (89·1 to 402·3)	16·9% (−25·7 to 94·7)
Metabolic risks			104 (70·4 to 148)	3·7 (2·5 to 5·3)	543 (370 to 758)	7·4 (5·0 to 10·3)	421·1% (297·2 to 588·3)	101·3% (54·2 to 165·3)	310 (214 to 436)	97·4 (66·4 to 137·5)	1610 (1110 to 2230)	196·2 (134·0 to 274·3)	418·9% (285·4 to 590·1)	101·5% (53·2 to 166·4)
	High fasting plasma glucose		41·5 (27·8 to 64·3)	1·5 (1·0 to 2·3)	228 (160 to 330)	3·2 (2·3 to 4·5)	450·7% (269·4 to 750·2)	106·4% (38·7 to 209·9)	117 (76·5 to 187)	38·0 (25·3 to 59·8)	664 (453 to 1020)	81·6 (56·7 to 120·2)	468·9% (260·3 to 821·8)	114·4% (41·4 to 235·0)
	High BMI		64·1 (32·3 to 106)	2·2 (1·1 to 3·7)	330 (150 to 520)	4·4 (2·0 to 7·0)	413·4% (270·2 to 599·0)	102·5% (45·4 to 175·5)	197 (106 to 321)	60·6 (31·4 to 99·9)	985 (496 to 1510)	119·8 (56·3 to 185·8)	398·9% (253·5 to 584·1)	97·7% (41·9 to 168·2)

Data in parentheses are 95% uncertainty intervals. DALY=disability-adjusted life-year.

Behavioural risks in 2023 accounted for the largest share of cancer deaths at 27·0% (95% UI 22·2–31·5), followed by metabolic risks at 5·9% (4·0–8·0) and environmental and occupational risks at 2·9% (2·0–3·8; GBD results tool).

The leading age-standardised death rate level 2 risk factors for both sexes combined in 1990 were unsafe sex, tobacco, dietary risks, and high alcohol use, whereas in 2023 they were unsafe sex, tobacco, dietary risks, and high BMI.

The proportion of cancer deaths attributable to risk factors was higher in females (38·1% [95% UI 31·3–44·6]) than males (27·1% [23·3–31·2]) in 2023 (GBD results tool). Among females, the leading age-standardised death rate level 2 risk factors contributing to cancer deaths were unsafe sex, dietary risks, high BMI, and tobacco use. In males, the leading risk factors were tobacco use, high alcohol consumption, dietary risks, and air pollution. The proportion of deaths attributable to risk factors increased only modestly over time—from 29·8% (25·7–34·4) in 1990 to 31·4% (27·1–35·7) in 2010 and 33·1% (28·7–37·2) in 2023—representing a 22·0% (2·0–48·6) overall rise in age-standardised risk-attributable cancer death rates from 1990 to 2023 (table 4; GBD results tool). In 2023, unsafe sex was the leading individual risk factor for cancer mortality in Africa, accounting for 108 000 (75 000–151 000) deaths, followed by tobacco use (72 000 [55 000–94 000]), dietary risks (50 000 [15 000–85 000]), and high body-mass index (33 000 [15 000–52 000]) in both sexes combined (table 4; appendix pp 192–199).

## Discussion

To our knowledge, this study is the first and most comprehensive longitudinal assessment of the cancer burden and its attributable risk factors in Africa over the past 33 years. Our analysis reveals a substantial increase in cancer incidence and mortality across the continent, with the burden disproportionately concentrated among children and adolescents and young adults. This escalating burden underscores the urgent necessity for targeted interventions and robust health-system strengthening. These findings provide a critical empirical framework to evaluate progress against Africa CDC strategic priorities,^17^ the WHO Global Action Plan for NCDs,^27^ and SDG 3.4.^15^ Furthermore, our results offer a foundational evidence base to guide policies and programmes aimed at mitigating the regional cancer burden and improving survival outcomes for the continent's most vulnerable populations.

Africa's cancer mortality trajectory diverges sharply from high-SDI and upper-middle-SDI regions.^21^ Although global age-standardised death rates have declined through improved prevention and care,^21^ Africa has experienced a doubling of absolute deaths and rising age-standardised mortality.^21^ This trend, characteristic of low-SDI settings,^21^, ^28^ reflects persistent barriers to screening, timely diagnosis, and treatment. Now the second leading cause of death in Africa after cardiovascular disease, cancer remains off-track to meet SDG 3.4—a one-third reduction in premature NCD mortality by 2030.^15^ These widening disparities necessitate urgent, equity-focused interventions, including strengthened surveillance, affordable diagnostics, and expanded treatment access.

About a third of cancer-related mortality and DALYs in Africa are attributable to modifiable risk factors identified in GBD 2023, a proportion notably lower than the global estimate of 42%.^21^ However, the low rate of cancer-related deaths and DALYs attributed to risk factors likely underestimates the true preventable burden, as current GBD frameworks exclude several oncogenic infections endemic to the continent; future iterations must incorporate these pathogens to refine risk-attributable estimates. Given that a substantial proportion of cancer mortality remains non-attributable to currently captured risks, evidence-based and effective strategies—integrating primary prevention with expanded early screening, diagnostic, and therapeutic capacity—are essential to mitigate the burgeoning cancer burden and improve survival outcomes across Africa.^21^

Cancer care in Africa is characterised by profound systemic inequities across the treatment continuum in terms of access, affordability, and safety. More than 80% of patients with cancer will require surgery during their illness,^29^ yet up to 95% of the population lacks access to safe and timely cancer surgery, compared with less than 5% in high-income regions.^30^ This disparity reflects systemic weaknesses: high out-of-pocket costs, shortages of trained personnel, inadequate infrastructure, and fragile governance. High-quality surgery is often confined to private institutions, excluding the majority of patients.^29^ *The Lancet Oncology* Commission has emphasised the urgent need for governments to invest in workforce development and publicly funded surgical systems, supported by regional centres of excellence and accreditation frameworks.^7^

Access to chemotherapy and radiotherapy is equally inadequate.^31^, ^32^ Chemotherapy, essential for curative and palliative care, remains inaccessible for most rural and vulnerable populations due to geographical and financial barriers. Radiotherapy is indispensable, with 50–70% of patients with cancer requiring it,^31^, ^32^ yet more than 90% of Africans lack access.^31^ *The Lancet Oncology* Commission on radiotherapy and theranostics highlights stark inequities between high-income and low-income countries, driven by machine shortages, long waiting times, and workforce deficits.^32^

Cancer patterns in Africa diverge substantially from global trends,^21^, ^25^ reflecting unique risk profiles and systemic inequities. Breast cancer in Africa, despite relatively low incidence, exhibits the highest mortality rates globally.^21^ Over three decades, incidence and mortality have risen concurrently in Africa, contrasting with declines in high-SDI regions.^21^ These disparities are rooted in late-stage presentation and constrained access to surgical, chemotherapeutic, radiotherapeutic, and rehabilitative services within Africa. Specialised oncology facilities remain scarce and urban-concentrated, while mammography, which is essential for early detection in high-SDI settings, is hindered by infrastructural and financial barriers across the continent.^33^ Such structural deficiencies in Africa affect the WHO Global Breast Cancer Initiative's targets.^12^

Similarly, cervical cancer remains the leading cause of female cancer mortality and DALYs in Africa, with the absolute burden doubling since 1990. Rising age-standardised rates underscore crucial gaps in human papillomavirus vaccination and screening. Fewer than 35% of African countries have implemented national vaccination programmes, and coverage remains below 50%, leaving the region off-track for WHO 2030 elimination targets.^11^ Reversing these trajectories for Africa requires equity-focused strategies that decentralise screening, expand access to multidisciplinary treatment, and scale up vaccination. Coordinated efforts are essential to ensure that progress towards global targets is inclusive and sustainable for the continent's most vulnerable populations

Gastrointestinal cancers represent a substantial and escalating burden in Africa, accounting for one-quarter of incident cases and DALYs, and a third of all cancer deaths in 2023, consistent with International Agency for Research on Cancer.^34^ These findings reflect an epidemiological transition; while infection-related malignancies such as stomach cancer have declined—largely due to improved *H pylori* management^35^, ^36^, ^37^ —metabolic and lifestyle-linked cancers, particularly colorectal and pancreatic, are increasing. Although colorectal cancer screening is cost-effective, its implementation is hindered by financial barriers, geographical inequities, and the lack of population-based recommendations for low-SDI settings.^38^, ^39^ Liver cancer remains a leading cause of mortality, primarily driven by hepatitis B virus (HBV) and hepatitis C virus in Africa. Despite the proven efficacy of birth-dose HBV vaccination, coverage in the WHO African region is only 18%, compared to 45% globally.^40^ Scaling up immunisation and maternal antiviral prophylaxis is essential to reducing vertical transmission. Addressing these disparities requires coordinated investment in diagnostic capacity, affordable treatment, and region-specific evidence to guide inclusive cancer control strategies.

Cancer burden in Africa demonstrates a striking age-related imbalance, with children, adolescents, and young adults contributing disproportionately to the overall burden.^41^ In 2023, childhood cancers in Africa represented about 38% of global cancer-related mortality and DALYs within this age group. Furthermore, malignancies in children and adolescents, and young adults accounted for 6·8% and 16·3% of total cancer deaths, respectively—figures that deviate sharply from global benchmarks of 1·0% and 4·6%.^21^ Conversely, mortality among adults aged 65 years and older comprised only 36·5% of the African total, contrasted with 61·7% globally.^21^ These divergent patterns reflect a combination of Africa's unique demographic structure and systemic inequities in access to timely diagnosis and curative treatment. These findings establish a crucial imperative for age-specific cancer-control strategies. Strengthening diagnostic infrastructure, expanding surgical and oncological capacity, and ensuring equitable access to chemotherapy and radiotherapy for children, adolescents, and young adults must be prioritised.^41^ Accelerated implementation of the CureAll framework and *The Lancet Oncology* Commission^7^ recommendations supported by sustained national government commitment and global partnerships, is essential to mitigate these inequities and safeguard the continent's future generations.^42^, ^43^

Despite a profound epidemiological transition since 2010, cancer control in Africa remains critically under-resourced,^44^ receiving only a fraction of the investment allocated to health care. National health-care expenditures consistently fail to meet international benchmarks; as of 2020, only Lesotho, Seychelles, and South Africa attained the 5% gross domestic product target established by the Addis Ababa Action Agenda,^45^ and the Abuja Declaration.^46^ This chronic underfunding is reflected in substantial out-of-pocket costs averaging 35·8%.^47^

Fiscal constraints are exacerbated by substantial governance cancer policy deficits;^48^, ^49^ about 37% of African nations lack a National Cancer Control Plan, while a further 26% possess plans that have expired.^50^, ^51^ The absence of active, domestically financed National Cancer Control Plans precludes the systematic adoption of cost-effective best buys and essential oncology packages recommended by the Disease Control Priorities (DCP-3), WHO, and *The Lancet Oncology* Commission.^7^ Reversing these trajectories requires a multisectoral shift towards sustainable domestic financing, augmented implementation capacity, and strategic international partnerships.

This study has some limitations inherent to GBD 2023 modelling and estimation within data-sparse environments. A fundamental constraint is the paucity of primary data; population-based cancer registries across Africa remain inconsistently distributed, predominantly urban-centric, or entirely absent in some countries. This necessitates a reliance on statistical interpolation and predictive covariates, which inherently widens uncertainty intervals for specific countries and malignancies. Furthermore, the characterisation of the non-fatal burden—specifically prevalence and YLDs—relies on the assumed geographic portability of US SEER parameters, scaled via mortality-to-incidence ratios. Such proxies might not fully capture Africa's distinct clinical landscape, which is characterised by late-stage presentation, limited screening and therapeutic infrastructure, and a high prevalence of comorbidities. This discrepancy potentially introduces systematic bias into disability intensity and survival duration estimates. Although the GBD framework's sophisticated modelling, covariates, and cross-validation techniques mitigate these deficiencies, these findings underscore the imperative for strengthened primary data infrastructure to refine future regional burden estimations. African nations are investing in robust health information systems at facility and population levels, while actively engaging in initiatives such as the Global Initiative for Cancer Registry Development to strengthen future cancer data.

In conclusion, the burden of cancer in Africa is large and increasing, with the burden disproportionately higher than that observed in high-income settings. Rising age-standardised death rate of cancer across the continent stands in stark contrast to the progress required to meet the Sustainable Development Goal target of reducing non-communicable disease mortality by a third between 2015 and 2030, as well as the African Union's Agenda 2063 commitments, further entrenching longstanding global health inequities. Given that about a third of cancer mortality and DALYs in Africa are attributable to modifiable risk factors, a harmonised, evidence-based, and continentally-led framework for primary prevention is urgently needed. The finding that Africa accounts for nearly 38% of global childhood cancer deaths underscores a profound inequity that demands coordinated regional and international action. Addressing systemic deficits across the cancer continuum—encompassing prevention, timely diagnosis, and equitable access to multimodal treatment—is imperative. These GBD estimates provide a robust empirical foundation upon which policy makers, funders, and international agencies must act to design context-specific, equity-driven strategies that curb the rising cancer burden and advance meaningful progress towards global health equity.

### GBD 2023 Africa Cancer Risk Factors Collaborators

### Affiliations

### Contributors

### Data sharing

GBD study 2023 data sources were available online from Global Health Data exchange tool (https://ghdx.healthdata.org/gbd-2023) and results tool (https://vizhub.healthdata.org/gbd-results/).

## Declaration of interests

We declare no competing interests.

## References

[1] WHO (Feb 3, 2020). WHO report on cancer: setting priorities, investing wisely and providing care for all. https://apps.who.int/iris/handle/10665/330745.

[2] Wild CP, Weiderpass E, Stewart BW (2020). World Cancer Report: cancer research for cancer prevention.

[3] Bray F, Laversanne M, Weiderpass E, Soerjomataram I (2021). The ever-increasing importance of cancer as a leading cause of premature death worldwide. Cancer.

[4] Bray F, Parkin DM, Gnangnon F (2022). Cancer in sub-Saharan Africa in 2020: a review of current estimates of the national burden, data gaps, and future needs. Lancet Oncol.

[5] Gelband H, Sankaranarayanan R, Gauvreau CL (2016). Costs, affordability, and feasibility of an essential package of cancer control interventions in low-income and middle-income countries: key messages from *Disease Control Priorities*, 3rd edition. Lancet.

[6] Foreman KJ, Marquez N, Dolgert A (2018). Forecasting life expectancy, years of life lost, and all-cause and cause-specific mortality for 250 causes of death: reference and alternative scenarios for 2016–40 for 195 countries and territories. Lancet.

[7] Ngwa W, Addai BW, Adewole I (2022). Cancer in sub-Saharan Africa: a *Lancet Oncology* Commission. Lancet Oncol.

[8] Sung H, Ferlay J, Siegel RL (2021). Global Cancer Statistics 2020: GLOBOCAN estimates of incidence and mortality worldwide for 36 cancers in 185 countries. CA Cancer J Clin.

[9] Meheus F, Atun R, Ilbawi A, Vaccarella S, Lortet-Tieulent J, Saracci R, Conway DI, Straif K, Wild CP (2019). Reducing social inequalities in cancer: evidence and priorities for research.

[10] Anorlu RI, Amaeshi LC, Okunade KS (2026). Cancer: a looming public health crisis in Africa-a call to accelerate equitable access to medicines. Oncologist.

[11] WHO Cervical Cancer Elimination Initiative. https://www.who.int/initiatives/cervical-cancer-elimination-initiative.

[12] WHO The Global Breast Cancer Initiative. https://www.who.int/initiatives/global-breast-cancer-initiative.

[13] WHO (2018). The Global Initiative for Childhood Cancer. Increasing access, advancing quality, saving lives. https://www.who.int/initiatives/the-global-initiative-for-childhood-cancer.

[14] Atun R, Bhakta N, Denburg A (2020). Sustainable care for children with cancer: a *Lancet Oncology* Commission. Lancet Oncol.

[15] UN (Oct 21, 2015). Transforming our world: the 2030 Agenda for Sustainable Development. https://www.un.org/ga/search/view_doc.asp?symbol=A/RES/70/1&Lang=E.

[16] African Union (September, 2015). Agenda 2063: the Africa we want. https://au.int/sites/default/files/documents/33126-doc-framework_document_book.pdf.

[17] Africa Centers for Disease for Disease Control and Prevention (Sept 2, 2023). Africa CDC strategic plan 2023–2027. https://africacdc.org/download/africa-cdc-strategic-plan-2023-2027/.

[18] GBD 2023 Disease and Injury and Risk Factor Collaborators (2025). Burden of 375 diseases and injuries, risk-attributable burden of 88 risk factors, and healthy life expectancy in 204 countries and territories, including 660 subnational locations, 1990–2023: a systematic analysis for the Global Burden of Disease Study 2023. Lancet.

[19] GBD 2023 Causes of Death Collaborators (2025). Global burden of 292 causes of death in 204 countries and territories and 660 subnational locations, 1990–2023: a systematic analysis for the Global Burden of Disease Study 2023. Lancet.

[20] Stevens GA, Alkema L, Black RE (2016). Guidelines for Accurate and Transparent Health Estimates Reporting: the GATHER statement. Lancet.

[21] GBD 2023 Cancer Collaborators (2025). The global, regional, and national burden of cancer, 1990–2023, with forecasts to 2050: a systematic analysis for the Global Burden of Disease Study 2023. Lancet.

[22] GBD 2021 Diseases and Injuries Collaborators (2024). Global incidence, prevalence, years lived with disability (YLDs), disability-adjusted life-years (DALYs), and healthy life expectancy (HALE) for 371 diseases and injuries in 204 countries and territories and 811 subnational locations, 1990–2021: a systematic analysis for the Global Burden of Disease Study 2021. Lancet.

[23] GBD 2019 Adolescent Young Adult Cancer Collaborators (2022). The global burden of adolescent and young adult cancer in 2019: a systematic analysis for the Global Burden of Disease Study 2019. Lancet Oncol.

[24] GBD 2021 Risk Factors Collaborators (2024). Global burden and strength of evidence for 88 risk factors in 204 countries and 811 subnational locations, 1990–2021: a systematic analysis for the Global Burden of Disease Study 2021. Lancet.

[25] Global Burden of Disease Cancer Collaboration (2019). Global, regional, and national cancer incidence, mortality, years of life lost, years lived with disability, and disability-adjusted life-years for 29 cancer groups, 1990 to 2017: a systematic analysis for the Global Burden of Disease Study. JAMA Oncol.

[26] GBD 2021 Demographics Collaborators (2024). Global age-sex-specific mortality, life expectancy, and population estimates in 204 countries and territories and 811 subnational locations, 1950–2021, and the impact of the COVID-19 pandemic: a comprehensive demographic analysis for the Global Burden of Disease Study 2021. Lancet.

[27] WHO (Nov 14, 2013). Global action plan for the prevention and control of noncommunicable diseases 2013–2020. https://apps.who.int/iris/bitstream/handle/10665/94384/9789241506236_eng.pdf?sequence=1&isAllowed=y.

[28] GBD 2019 Asia and All Cancers Collaborators (2024). Temporal patterns of cancer burden in Asia, 1990–2019: a systematic examination for the Global Burden of Disease 2019 study. Lancet Reg Health Southeast Asia.

[29] Sullivan R, Alatise OI, Anderson BO (2015). Global cancer surgery: delivering safe, affordable, and timely cancer surgery. Lancet Oncol.

[30] Alkire BC, Raykar NP, Shrime MG (2015). Global access to surgical care: a modelling study. Lancet Glob Health.

[31] Mallum A, Sibanda W, Tendwa MB (2024). An insight into the current status of the radiotherapy landscape in Africa: a cross-sectional survey study on the available infrastructure and human resources. Med Res Arch.

[32] Abdel-Wahab M, Giammarile F, Carrara M (2024). Radiotherapy and theranostics: a *Lancet Oncology* Commission. Lancet Oncol.

[33] Mo T, Joffe M, Cubasch H (2025). Breast cancer overall survival, annual risks of death, and survival gap apportionment in sub-Saharan Africa (ABC-DO): 7-year follow-up of a prospective cohort study. Lancet Glob Health.

[34] International Agency for Research on Cancer Global burden of gastrointestinal cancers. https://gco.iarc.who.int/stories/gastro-intestinal/en.

[35] Howson CPHT, Hiyama T, Wynder EL (1986). The decline in gastric cancer: epidemiology of an unplanned triumph. Epidemiol Rev.

[36] Pagano JS (1999). Epstein-Barr virus: the first human tumor virus and its role in cancer. Proc Assoc Am Physicians.

[37] Yin H, Qu J, Peng Q, Gan R (2019). Molecular mechanisms of EBV-driven cell cycle progression and oncogenesis. Med Microbiol Immunol.

[38] Zhang L, Mosquera I, Lucas E (2023). CanScreen5, a global repository for breast, cervical and colorectal cancer screening programs. Nat Med.

[39] Martinez ME, Schmeler KM, Lajous M, Newman LA (2024). Cancer screening in low- and middle-income countries. Am Soc Clin Oncol Educ Book.

[40] Riches N, Henrion MYR, MacPherson P (2025). Vertical transmission of hepatitis B virus in the WHO African region: a systematic review and meta-analysis. Lancet Glob Health.

[41] GBD 2023 Childhood Cancer Collaborators (2026). Global burden of cancer in children and adolescents aged 0–19 years, 1990–2023: a systematic analysis for the Global Burden of Disease Study 2023. Lancet.

[42] Ortiz R (2024). WHO Global Initiative for Childhood Cancer Countries Engaged. https://siop-online.org/wp-content/uploads/2024/05/GICC-2024-Updated-Levels-of-Country-Engagement-V2.pdf.

[43] WHO (Oct 28, 2021). CureAll framework: WHO global initiative for childhood cancer: increasing access, advancing quality, saving lives. https://iris.who.int/bitstream/handle/10665/347370/9789240025271-eng.pdf?sequence=1.

[44] Jailobaeva K, Falconer J, Loffreda G, Arakelyan S, Witter S, Ager A (2021). An analysis of policy and funding priorities of global actors regarding noncommunicable disease in low- and middle-income countries. Global Health.

[45] UN (2015). Addis Ababa Action Agenda of the Third International Conference on Financing for Development (Addis Ababa Action Agenda). https://www.un.org/esa/ffd/wp-content/uploads/2015/08/AAAA_Outcome.pdf.

[46] Organisation of African Unity (2001). Declaration on HIV/AIDS, tuberculosis and other related infectious diseases. https://au.int/sites/default/files/pages/32894-file-2001-abuja-declaration.pdf.

[47] WHO (2024). WHO African Region Health Expenditure Atlas 2023. https://www.afro.who.int/publications/who-african-region-health-expenditure-atlas-2023-0.

[48] WHO (2002). National cancer control programmes: policies and managerial guidelines, 2nd ed. https://apps.who.int/iris/handle/10665/42494.

[49] Romero Y, Trapani D, Johnson S (2018). National cancer control plans: a global analysis. Lancet Oncol.

[50] International Cancer Control Partnership National plans. https://www.iccp-portal.org/map.

[51] Garton EM, Ali Z, Cira MK (2023). An analysis of survivorship care strategies in national cancer control plans in Africa. J Cancer Surviv.

